# The “Vesicular Intelligence” Strategy of Blood Cancers

**DOI:** 10.3390/genes12030416

**Published:** 2021-03-13

**Authors:** Dorian Forte, Martina Barone, Francesca Palandri, Lucia Catani

**Affiliations:** 1 IRCCS Azienda Ospedaliero—Department of Experimental, Diagnostic and Specialty Medicine, School of Medicine, Institute of Hematology “Seràgnoli”, University of Bologna, 40138 Bologna, Italy; dorian.forte2@unibo.it (D.F.); martina.barone5@unibo.it (M.B.); 2 IRCCS Azienda Ospedaliero—Institute of Hematology “Seràgnoli”, University of Bologna, 40138 Bologna, Italy

**Keywords:** blood cancers, extracellular vesicles, disease biomarker, bone marrow microenvironment, angiogenesis, hypercoagulability, immune evasion, drug resistance

## Abstract

Blood cancers are a heterogeneous group of disorders including leukemia, multiple myeloma, and lymphoma. They may derive from the clonal evolution of the hemopoietic stem cell compartment or from the transformation of progenitors with immune potential. Extracellular vesicles (EVs) are membrane-bound nanovesicles which are released by cells into body fluids with a role in intercellular communication in physiology and pathology, including cancer. EV cargos are enriched in nucleic acids, proteins, and lipids, and these molecules can be delivered to target cells to influence their biological properties and modify surrounding or distant targets. In this review, we will describe the “smart strategy” on how blood cancer-derived EVs modulate tumor cell development and maintenance. Moreover, we will also depict the function of microenvironment-derived EVs in blood cancers and discuss how the interplay between tumor and microenvironment affects blood cancer cell growth and spreading, immune response, angiogenesis, thrombogenicity, and drug resistance. The potential of EVs as non-invasive biomarkers will be also discussed. Lastly, we discuss the clinical application viewpoint of EVs in blood cancers. Overall, blood cancers apply a ‘vesicular intelligence’ strategy to spread signals over their microenvironment, promoting the development and/or maintenance of the malignant clone.

## 1. Introduction

Blood cancers are a heterogeneous group of disorders including leukemia, multiple myeloma, and lymphoma. They may derive from the clonal evolution of the hemopoietic stem cell compartment or from the transformation of progenitors with immune potential. The functional cross-talk between blood cancer cells and the surrounding microenvironment is of utmost importance for the development and maintenance of the malignant cells. However, despite the huge interest in the tumor microenvironment, unexplored microenvironmental-driven mechanisms and signals in blood cancers still need to be defined.

Intriguingly, in addition to cell–cell contact and soluble signals, extracellular vesicle (EV) generation/release from blood cells and/or microenvironment niche has been described as the rising successful strategy for complex intercellular communication in tumors, including blood cancers [1]. In fact, sending information enclosed in a plasma membrane represents a “smart strategy” to avoid degradation and promote intercellular signaling, not only in physiology but also in cancer. Notably, the EV-driven “smart strategy” assures blood cancer cells’ functional activity and survival and the generation of a niche with cancer-promoting effects. In other words, since strategic blood cancer priority is the spreading of tumor signals, the EVs represent a “sustainable” and effective biological tool by which these requirements are pursued. The marrow niche and the immune and coagulation systems have been depicted as main areas for strengthening synergies and collaboration in blood cancers through EV-based communication.

Several studies have identified EVs as delivery vehicles of blood cancer-released components in peripheral blood, highlighting their clinical relevance in diagnosis and prognosis. Of note, in precision medicine, EVs might be considered a further promising tool for liquid biopsy in monitoring disease progression. Moreover, due to their biological properties and function, EVs have drawn attention not only as a potential therapeutic target but also as drug delivery vehicles [2,3,4,5,6,7,8].

**Figure 1:**
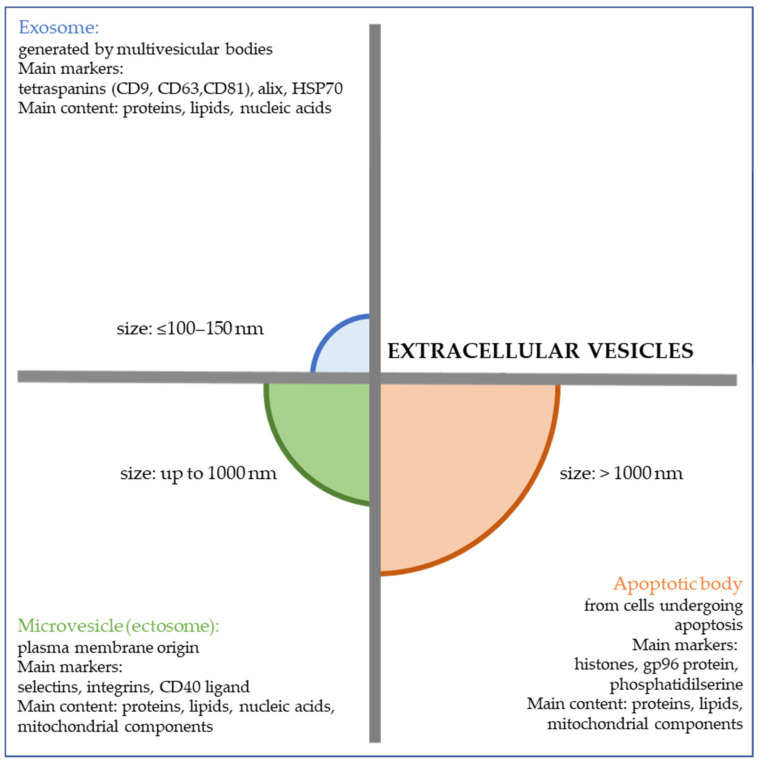
Main characteristics of the three major subpopulations of extracellular vesicles: exosomes, microvesicles, and apoptotic bodies in terms of size, biogenesis, main markers, and content [9,13,14,20].

EVs are released from various cells during homeostasis and cell activation, with pleiotropic effects on signaling among cells. They have been detected in several biological fluids, including plasma, urine, and saliva. The same cells may serve as recipients or effectors of EV targeting, and various mechanisms of release or uptake have been previously described [9]. EV cargos are enriched in nucleic acids, proteins, and lipids, and studies focusing on EV cargo packaging under different conditions have been reported in public databases, including ExoCarta [10], Vesiclepedia [11], and EVpedia [12]. Briefly, the International Society of Extracellular Vesicles has classified EVs into three main groups: (1) exosomes, small vesicles with diameters ≤100–150 nm that are formed inside multivesicular bodies; (2) microvesicles, medium-size vesicles of plasma membrane origin with diameters of up to 1000 nm; and (3) apoptotic bodies, large vesicles with diameters > 1000 nm that are produced by apoptotic cells [13] (Figure 1). Excellent reviews on the biomolecular and functional characteristics of EVs as well as on the techniques used in EV isolation and characterization have recently been published [9,10,11,12,13,14]. EVs affect normal and malignant hemopoiesis and are critical players in the regulation of inflammation and immunity [15,16,17,18]. Specifically, blood cancer-derived EVs deeply impact stromal cell transformation, angiogenesis, and immune suppression by carrying mRNAs, microRNA (miR), lipids, and proteins and transferring the cargo to target cells including stromal cells, endothelial cells, and immune cells. Usually, these effects aim to promote malignant transformation, development, and progression [19]. Therefore, the importance of EVs in these hematologic disorders is mainly attributed to their role in changing the tumor microenvironment, inducing tumor immune evasion and chemoresistance, and driving the hypercoagulable state.

In this review, we will describe the “smart strategy” on how blood cancer-derived EVs regulate tumor cell development and spreading, thrombogenicity, immune response, drug resistance, and communication within the tumor microenvironment in blood cancers. Overall, blood cancers simulate human intelligence by creating a ‘vesicular intelligence’ strategy to spread signals over their microenvironment. The potential utility of EVs as non-invasive biomarkers will be also discussed. Indeed, EV cargo can mirror blood cancer cell complexity and enable the measurement of multiple biological components shed by malignant cells. Moreover, we will summarize the heterogeneous roles of microenvironment-derived EVs in various blood cancers. Note that, throughout this review, the term EVs refers to both exosomes and microvesicles.

We identified references for this review via a review of literature in PubMed using a Boolean search strategy with the terms “extracellular vesicles”, “exosomes”, “microRNA”, “blood cancer”, “hematologic malignancy”, “leukemia”, “lymphoma”, “myeloma”, “myelodysplastic syndrome”, and “myeloproliferative neoplasms”. We identified additional articles from reference lists of the selected articles and through expert reviews of the literature. We included all English-language articles published from database inception through to November 2020.

## 2. The Secret Signature of the Blood Cancer-Derived EVs

Recently, the number as well as the cargo, including proteins, microRNA (miR), and long non-coding RNA (lncRNA), have been reported to be upregulated in the EVs of patients with blood cancers, suggesting that circulating EVs might be a diagnostic marker for these disorders. Indeed, due to their location in blood, EV-based diagnostics may represent an optimal candidate for non-invasive diagnosis. Further, by analyzing deregulated EV cargo, a tool for the rapid diagnosis of disease relapse and the selection of the most appropriate personalized therapy can be available. However, although research has demonstrated EVs’ usefulness, the challenge is still to establish and integrate EV-derived biomarkers in the clinic.

One of the first studies, conducted by Caivano et al. [21], demonstrated that circulating EVs may represent a novel biomarker, since high serum levels of EVs are detected in the peripheral blood of patients with various types of blood cancers. They found EVs to be elevated in acute myeloid leukemia (AML), multiple myeloma (MM), Hodgkin lymphoma (HL), Waldenstrom Macroglobulinemia (WM), and some myeloproliferative neoplasms (MPN); besides this, EVs from HL, MM, and MPN were characterized by a lower size. Notably, EVs specifically expressed cancer-related antigens (e.g., CD19 in B-cell neoplasms, CD38 in MM, CD13 in myeloid tumors, CD30 in HL), revealing that the total and antigen-specific count of EVs correlated with the clinical features.

### 2.1. Acute Myeloproliferative Disorders

Circulating EVs might be a diagnostic and prognostic marker for AML. Indeed, since AML EVs express membrane proteins of blast cells, they might be biomarkers of leukemia dynamics and the presence of minimal residual disease. Notably, miR EV content has been suggested to be a distinctive feature.

Szczepanski et al. demonstrated that sera from newly diagnosed AML patients contained higher levels of EVs compared to their normal counterparts [22]. Notably, isolated EVs have a distinct molecular profile: in addition to conventional EV markers, they contain membrane-associated transforming growth factor (TGF)-β1; MICA/MICB; and myeloid blasts markers such as CD34, CD33, and CD117. Interestingly, the cargo protein TGF-β1 has been suggested as a potential biomarker for AML patients undergoing post-chemotherapy consolidation supportive therapy [22,23]. Monitoring newly diagnosed AML patients (diagnosis, nadir, remission), Tzoran I et al. demonstrated that the total circulating EV numbers were higher in patients in the first remission compared with controls [22]. Of note, at all three time points, the endothelial EV proportion was higher compared with controls. Relapse remains the major cause of mortality for patients with AML. Current techniques detect circulating blasts that coincide with advanced disease and poorly reflect minimal residual disease during early relapse. Recently, serum exosome miRs have been proposed as a platform for a novel, sensitive biomarker for the prospective tracking and early detection of AML recurrence. Specifically, the combined detection of miR-150, -155, and -1246 in AML-derived EVs was proposed as a marker to monitor patients following treatment [24]. Furthermore, EV-miR-125b or EV-miR-10b might also serve as a promising marker for predicting the prognosis of AML patients [25,26].

### 2.2. Chronic Lympho/Myeloproliferative Disorders

Accumulating evidence highlights circulating EVs as potential biomarkers of CLL disease stages. It has been firstly demonstrated that the total circulating EV level in CLL was significantly higher compared with healthy subjects and that patients’ EVs exhibit a phenotypic shift from predominantly platelet-derived in the early stage to leukemic B-cell-derived at an advanced stage. Additionally, CD19+ and CD37+ B-cell-derived EVs significantly correlate with a high tumor burden [27,28].

Regarding protein content, Belov et al. [29] showed that CD19+ EVs from the plasma of CLL patients express a subset (~40%) of proteins detected on CLL cells from the same patients: moderate or high levels of CD5, CD19, CD31, CD44, CD55, CD62L, CD82, HLA-A, B, C, HLA-DR; low levels of CD21, CD49c, CD63. Different proteomic profiles of plasma exosomes have been demonstrated between indolent and progressive CLL, as well as within individual patients at the time of disease onset and during evolution. High levels of S100-A9 protein, a molecule involved in cell cycle regulation, have been observed also in plasma-derived exosomes from patients with progressive CLL compared to indolent CLL patients, highlighting the importance of exosomes as mediators of CLL progression [30].

Interestingly, the dynamic accumulation of CD52+ EV in plasma can be used to study CLL progression. To this purpose, Boysen et al. showed that CLL B-cells generate EVs spontaneously and that EVs released can be further induced by B-cell receptor [BCR]-ligation, while an increased accumulation of CD52+ EV is detected in previously untreated CLL patients with progressive disease [31].

Yeh et al. [32] recently reported that CLL cells release significant amounts of exosomes in plasma with abundant CD37, CD9, and CD63 expression. Interestingly, the miR analysis of plasma-derived exosomes identified a distinct miR signature, including miR-29 family, miR-150, miR-155, and miR-223, which have been associated with CLL. This study also highlights the regulation of BCR signaling in the release of CLL exosomes: BCR activation by α-immunoglobulin (Ig) M induces exosome secretion, whereas ibrutinib-driven BCR inactivation prevents α-IgM-stimulated exosome release and significantly decreases the exosome plasma concentration. Moreover, exosomes released by CLL cells are enriched by miR-202-3p [33]. Finally, Wu Z. et al. prove that mc-COX2 (a critical mitochondrial-circRNA highly expressed in plasma), delivered by CLL exosomes, is associated with leukemogenesis and poor prognosis in CLL patients [34].

In the setting of MPN, we recently demonstrated that an increased/decreased proportion of circulating platelet (CD61+CD62P+)-/megakaryocyte (CD61+CD62P-)-EVs is observed in patients with myelofibrosis (MF) and essential thrombocythemia (ET). Additionally, the JAK1/2 inhibitor Ruxolitinib normalizes the profile of circulating EVs in the MF spleen-responder patients only by increasing the megakaryocyte EVs and decreasing the platelet EV proportions. Importantly, a cut-off value of 19.95% for megakaryocyte-derived EVs identifies spleen responders and non-responders, demonstrating that circulating megakaryocyte EVs, as a liquid biopsy assay, might be a potential tool to predict response to ruxolitinib therapy in MF [35]. Additionally, in plasma-derived EVs from MF patients, we identified a distinct miR profile and mitochondrial components, suggesting EVs as potential markers of aggressive disease, especially in triple-negative MF patients [36].

Moreover, in systemic mastocytosis, it has been demonstrated that serum from patients contains EVs with a mast cell signature, and their concentrations correlate with surrogate markers of disease [37]. Finally, it has also been demonstrated that EVs from CML CD34+ cells are associated with an increase in immature cells in the peripheral blood [38].

### 2.3. Multiple Myeloma

In MM, EVs expressing CD38, CD138, CD44, and CD147 allowed the stratification of patients by disease phase and therapy response [39]. Consistently, it has been reported that CD138+ circulating EVs are elevated across all stages of disease and mirror plasma-cell burden and treatment response [40]. Once again, serum exosomal miR can be used independently as a novel biomarker. The levels of serum exosome-derived miR-20a-5p, miR-103a-3p, and miR-4505 were significantly different among patients with MM, patients with smoldering myeloma (SMM), and healthy individuals, while differences in the levels of let-7c-5p, miR-185-5p, and miR-4741 discriminated MM patients from SMM patients or healthy controls [41]. Additionally, it was found that let-7b and miR-18a were significantly associated with both progression-free survival and overall survival in newly diagnosed MM patients [42]. Furthermore, when investigating the lncRNA expression profile of serum exosomes, only one exosomal lncRNA—a psoriasis susceptibility-related RNA gene induced by stress (PRINS)—was found to be differentially expressed in MM vs. healthy donors, suggesting its possible diagnostic role [43].

### 2.4. Lymphoma

Lymphoma cell-derived EVs isolated from non-HL patients are enriched in CD19 and CD20, while EVs isolated from patients with HL are enriched in CD30. Therefore, CD30+ lymphoma cell-derived EVs might have a diagnostic and prognostic role. The number of specific lymphoma cell-derived EVs and their surface markers also correlate with lymphoma subtypes [21]. Additionally, it has been described that EVs carry tumor antigens and express cancer cell-derived molecules, such as CD19, CD20, and CD22, which may be involved in the cell-to-cell communication of the lymphoma microenvironment [44]. It was first proposed that CD20+ lymphoma cell-derived EVs are the best biomarkers for disease progression and antibody-based treatment response, as their circulating level directly correlates with CD20+ circulating cells in patients [45]. Consistently, a direct correlation between circulating lymphoma cell-derived EV number, disease progression, and response to treatment have been reported. Specifically, Van Eijndhoven et al. [46] demonstrated that the isolated EV fractions of untreated classical HL patients show enriched levels of miR-24-3p, miR-127-3p, miR-21-5p, miR-155-5p, and let-7a-5p. Monitoring EV miRNA levels in patients before treatment, after treatment, and during long-term follow-up demonstrated a stable reduction in miR levels, matching a complete metabolic response, as observed with FDG-PET. Importantly, EV miR levels rose again in relapsed patients. Interestingly, the feasibility of monitoring cancer progression by evaluating B-cell lymphoma 6 (BCL-6) and c-Myc mRNA levels in EVs isolated from the plasma of patients with B-cell lymphomas has recently been demonstrated. The study demonstrated that both markers are predictors of worse outcome [47].

Overall, growing evidence highlights serum/plasma EVs as potential biomarkers with diagnostic and prognostic implications in acute and chronic blood cancers (Supplementary Materials Table S1). In particular, in the attempt to identify circulating EVs as disease-stage biomarkers, significant associations have been found between EV biomolecular cargo and disease activity/progression. However, although these studies have prompted the clinical application of EVs in blood cancers, some problems need to be further elucidated. Firstly, which component of EVs may be the most suitable for biomarker identification still needs to be addressed. Secondly, there is a lack of reliable methods for practical and reproducible application in the clinic.

## 3. Re-Education of the Bone Marrow Niche: Lessons from EVs

Recent experimental evidence and clinical findings support the concept that, in blood cancers, the interactions between tumor cells and bone marrow microenvironment are regulated, at least in part, by EVs [48,49,50,51]. This “vesicular intelligence strategy” is driven by a bi-directional cross-talk where malignant EVs modify the bone marrow niche in favor of blood cancer cells at the expense of the normal hemopoietic stem cells by reducing the antineoplastic immunity and promoting resistance to therapy. It is therefore of the utmost importance to discover and unravel this tumor-stroma interaction and the underlying mechanisms to develop effective therapeutic strategies [52].

### 3.1. EVs Derived from AML Cells

Recent studies have addressed the role of EVs from leukemic cells in shaping the AML bone marrow niche. Leukemia cells manipulate the bone marrow microenvironment, partly through leukemia-derived EVs, to suppress normal hemopoiesis and facilitate the growth of leukemic counterparts [53].

Consistently, Kumar et al. [54] demonstrated that engrafted AML cells or AML-derived EVs increase mesenchymal stromal progenitors and block osteolineage development and bone formation in vivo. Indeed, AML-derived EVs induce the downregulation of hematopoietic stem cell-supporting factors such as KIT ligand (KITL), C-X-C motif chemokine ligand (CXCL)12, and insulin growth factor (IGF)-1 in bone marrow stromal cells and reduce their ability to support normal hemopoiesis. Horiguchi et al. [55] firstly demonstrated that, in addition to AML-EV, myelodysplastic syndrome (MDS) EVs are also linked to stromal cell dysfunction.

Coding and non-coding RNAs seem to play a key role in the process of bone marrow environment conditioning by leukemic cells. It has been reported that exosomes from primary AML cells and AML cell lines contain miR-155, miR-375, and miR-150. These miRNAs modulate the secretion of cytokines and growth factors by cells of the bone marrow microenvironment as well as the proliferation and migration of hemopoietic stem cells by affecting the CXCR4/SDF-1 axis, which plays a pivotal role in the retention and differentiation of hemopoietic stem cells in the bone marrow [56]. Then, it has been demonstrated that AML and MDS cells reduce the hemopoiesis-supportive capacity of the mesenchymal stromal cells (MSC) by delivering miR-7977 via EVs. Mechanistically, the miR-7977 cargo of the AML/MDS EVs contributes to the decrease in hematopoietic supportive factors, including Jagged-1, stem cell factor (SCF), and angiopoietin-1, in AML cells [57].

Finally, it has been recently demonstrated that secretory proteins from hematopoietic stem cells undergo exosomal maturation and release under the control of vacuolar protein sorting protein 33b (VPS33B). Specifically, VPS33B is highly expressed in both hematopoietic stem cells and leukemia-initiating cells and is involved in exosome maturation and secretion to maintain their stemness by regulating the release of selected growth factors such as Thrombopoietin (TPO) and Angiopoietin Like (ANGPTL) 2 and 3. Interestingly, VPS33B deficiency led to a delayed onset of leukemogenesis [58].

### 3.2. EVs Derived from CLL Cells

Multiple studies have shown that CLL cells are dependent on their microenvironment for survival. The interplay between CLL cells and the microenvironment is mediated through direct cell contact and soluble factors, as well as EVs. Consistently, a bi-directional cross-talk has been reported between CLL B-cells and MSC via EVs. MSC-derived EVs increase the migration and survival of CLL B-cells and change their gene expression profile [59]. CLL B cells-derived EV, in turn, can transfer miR, promoting the migration, survival, and proliferation of MSCs [31,32,58]. Ghosh et al. [27] found that circulating CLL-EVs can activate the AKT signaling pathway in CLL-bone marrow stromal cells with the production of vascular endothelial growth factor (VEGF), a survival factor for CLL B cells, highlighting the important role of EVs in the activation of the tumor microenvironment. In addition, it has been described that CLL-derived exosomes enriched with miR-146a and miR-451 may induce the transition of stromal cells toward cancer-associated fibroblasts. Once again, leukemic EVs create a favorable environment to promote CLL progression [60,61].

### 3.3. EVs Derived from CML Cells

Corrado C et al. demonstrated that exosomes from CML cells promote the proliferation and survival of leukemic cells, both in vitro and in vivo, by inducing interleukin (IL)-8 secretion from stromal cells. In turn, IL-8 or LAMA84 (a human chronic myeloid leukemia cell line) -conditioned medium increases CML cells’ motility as well as ability to adhere to a monolayer of bone marrow stromal cells [60,62]. It has been also reported that K562-EVs enhance the proliferation and increase the expression of BCR-ABL1 in bone marrow MSC, which, in turn, increases the secretion of TGF-β1. Notably, these bone marrow MSC trigger the TGF-β1-dependent proliferation of K562 cells [63]. Very recently, the role of the miR cargo of CML EVs in microenvironment transformation has been addressed. Interestingly, K562 cell-derived exosomal miR-711 can be transferred to bone marrow MSCs and weaken their adhesive abilities by inhibiting the expression of the adhesion molecule CD44 [64]. Additionally, Gao et al. [65] reported that CML cells remodel the bone marrow niche via the exosome-mediated transfer of miR-320, indirectly promoting leukemia progression. In detail, miR-320-enriched EVs are endocytosed by bone marrow MSC and thus inhibit osteogenesis.

### 3.4. EVs Derived from MM Cells

Previous studies demonstrated how MM cells affect the bone marrow microenvironment (especially MSCs and other stromal cells) to promote cancer progression and survival through the secretion of soluble factors and differentiating them into cells that support the expansion of MM cells [66,67,68]. To this purpose, EVs may play a key role in the cross-talk between MM cells and MSC/bone marrow stromal cells and may be a potential therapeutic target.

In MM, the balance between osteoclasts and osteoblasts activity is lost in favor of osteoclasts, thus resulting in skeletal disorders. Consistently, it has been demonstrated that exosomes derived from MM patients’ sera promote osteoclast function and differentiation [69]. It has also been shown that MM EVs promote IL-6 secretion and suppress the osteoblastic differentiation and mineralization of bone marrow MSC [70]. Faict et al. [71] recently demonstrated that MM EVs not only enhance osteoclast activity but also block osteoblast differentiation and functionality in vitro. Interestingly, blocking exosome secretion using the sphingomyelinase inhibitor GW4869 sensitizes MM cells to bortezomib [72].

The EV cargo of coding and non-coding RNA seems to have a relevant role in the mechanisms of osteoblast/clastogenesis. A key role of exosomal lncRUNX2-AS1 from MM cells in the osteogenic differentiation of MSCs has been recently demonstrated. Specifically, bioactive lncRNA RUNX2-AS1 in MM cells might be packed into exosomes and delivered to MSCs, thus repressing the osteogenesis of MSCs [73]. Umezu et al. [74] demonstrated that EV-encapsulated miR-10a expression was high, while intracellular miR-10a was low in MM bone marrow stromal cells. Of note, the inhibition of EV release resulted in the accumulation of intracellular miR-10a, the inhibition of cell proliferation, and the promotion of apoptosis in MM bone marrow stromal cells. Furthermore, when miR-10a derived from MM bone marrow stromal cells was transferred into MM cells via EVs, the proliferation of MM cells was enhanced. These results suggest that miR-10a might have a regulatory role in both the bone marrow microenvironment and MM cells. Interestingly, EVs derived from MM cells transfer miR-146a into MSCs, inducing the secretion of elevated levels of cytokines, which promote both MM cell viability and migration. Specifically, enriched miR-146a MSCs show increased cytokine/chemokine secretion (including CXCL1, IL6, IL8, IP-10, MCP-1, and C-C motif ligand (CCL)-5), which in turn favors MM cell growth and migration [75]. Accordingly, Cheng et al. demonstrated that exosomes secreted by the MM cell line OPM2 in vitro are enriched in miR-146a and miR-21, which can increase the IL-6 production, cell proliferation, and cancer-associated fibroblast transformation of MSCs after co-culture with OPM2-conditioned media [76]. Finally, it has been recently demonstrated that MM cell-derived exosomes induce the overexpression of miR-27b-3p and miR-214-3p in fibroblasts, increasing their proliferation and resistance to apoptosis. Accordingly, these data support an active role of MM cells in creating a supportive bone marrow microenvironment [77].

### 3.5. EVs Derived from Lymphoma Cells

EVs seem to be a novel communication mechanism between lymphoma cells and their microenvironment, playing a role in lymphomagenesis. This might include a bi-directional cross-talk between malignant cells and resident fibroblasts. EVs alter the phenotype of fibroblasts to support tumor growth and exert a role in the establishment of the tumor-promoting microenvironment in HL. Specifically, EVs collected from HL cells are internalized by fibroblasts and trigger their migration ability and an inflammatory phenotype. In turn, EV-treated fibroblasts release pro-inflammatory cytokines, growth factors, and pro-angiogenic factors, which are known to promote HL progression [78]. It is noteworthy that it has been described that CD30+ EVs bind to CD30L on bystander cells and present additional membrane-associated CD30 sites for the binding and toxic activity of Brentuximab-Vedotin, suggesting the dual targeting of cancer and bystander cells [79].

Overall, these findings contribute to explaining how the bone marrow niche becomes re-educated by EVs from blood cancers (Supplementary Materials Table S2). The fine interplay between malignant cells and their microenvironment is deeply altered in these disorders, and EVs seem to have a relevant impact on it. Most of the information points to the impact of EVs on microenvironment damage, spreading inflammatory signals, and altering adhesive functions.

## 4. Promotion of Blood Cell Malignancy by EVs from the Bone Marrow Microenvironment

The goal of cancer cells is to develop a sustainable and efficient strategy that assures their survival, maintenance, and spreading. For this purpose, in the setting of blood cancers, protective signaling from the microenvironment promotes leukemia cell persistence, the development of chemoresistance, and disease relapse. Direct evidence of the involvement of EVs derived from cells of the bone marrow microenvironment in promoting the survival and functional behavior of malignant cells is progressively growing.

### 4.1. Acute Myeloproliferative Disorders

It has been demonstrated that fibroblast growth factor 2 (FGF2)-enriched exosomes from bone marrow stromal cells are endocytosed by leukemia cells (AML) and protect leukemia cells from tyrosine kinase inhibitors (TKIs). The expressions of FGF2 and its receptor, FGFR1, are both increased in a subset of stromal cell lines and primary AML stroma, and the increased FGF2/FGFR1 signaling is associated with enhanced exosome secretion. Interestingly, the inhibition of FGFR in vitro and in vivo can reduce exosome secretion and regulate stromal function, and might be a therapeutic option to overcome resistance to TKIs [80].

### 4.2. Chronic Lympho/Myeloproliferative Disorders

EVs from the MSCs of MDS patients modify CD34+ cell properties, contributing to the maintenance of clonal hemopoiesis. Specifically, miR-10a and miR-15a are overexpressed in EVs from MDS MSCs. These microRNAs are transferred to CD34+ cells through EVs, promoting cell viability and clonogenic capacity and altering their miRs and gene expression [81]. Similarly, EVs released by the MSC of patients with MPN, enriched in miR-155, promote the clonogenic ability of the malignant CD34+ cells [82]. The clonal hemopoiesis of CML is sustained by leukemia stem cells. miR-126 is necessary for normal and leukemic stem cell quiescence and self-renewal. It has recently been demonstrated that bone marrow endothelial cells supply miR-126 to CML leukemic stem cells through EV release to support quiescence and leukemia growth. Specifically, BCR-ABL-mediated SPRED1 phosphorylation decreases miR-126 biogenesis in CML leukemic stem cells. The consequence is that quiescence and the leukemogenic ability of CML leukemic stem cells rely only on the EV-based trafficking of miR-126 from endothelial cells to leukemic stem cells in the bone marrow niche. Consistently, TKI therapy inhibits BCR-ABL-induced SPRED1 phosphorylation, leading to an increase in miR-126 levels. Notably, miR-126 KO or treatment with a CpG-miR-126 inhibitor targeting miR-126 in both leukemic stem cells and endothelial cells increases the in vivo anti-leukemic effects of TKI treatment and highly reduces the leukemia-initiating ability of leukemic stem cells [83].

EVs play also a crucial role in CLL B cells/bone marrow microenvironment communication. Co-cultures of EVs from CLL bone marrow MSCs with CLL B cells results in a decrease in leukemic cell apoptosis and migration ability and an increase in their chemoresistance to selected drugs, including fludarabine, ibrutinib, idelalisib, and venetoclax [59].

### 4.3. Multiple Myeloma

The bone marrow stromal cells of MM patients secrete exosomes with an altered composition compared to those produced by their normal counterparts. Lower levels of the oncosuppressor miR-15a promote the proliferation of MM cells. In addition, MM bone marrow MSC-derived exosomes show higher levels of oncogenic proteins, cytokines, and adhesion molecules compared with their cells of origin. Importantly, whereas MM bone marrow MSC-derived exosomes promote MM tumor growth, normal bone marrow MSC exosomes inhibit the growth of MM cells [84]. Consistently, it has been described that, in contrast to EVs from MM patients, EVs from the bone marrow MSCs of healthy donors decrease the viability, proliferation, and migration of MM cells via the activation of a mitogen-activated protein kinase (MAPK) pathway [85]. Recently, it has been also highlighted that long non-coding RNAs from cells of the bone marrow microenvironment are also involved in the regulation of MM development. To this purpose, it has been demonstrated that LINC00461, a sponge for miR-15a/16, is highly expressed in MSC-derived exosomes and increases MM cell proliferation by regulating miR15a/16 and BCL-2 [86].

Altogether, these observations reinforce the importance of EVs from key cells of the bone marrow microenvironment (such as MSCs and endothelial cells) in promoting and favoring the malignant cells of blood cancers (Supplementary Materials Table S3). Whether this is a primary event or is secondary to the EV-driven signals of malignant cells to the bone marrow microenvironment remains a matter of discussion. Anyway, these findings suggest that therapeutic attempts should target not only the EV-driven strategy of the malignant cells but also its microenvironmental counterparts.

## 5. Blood Cancer Progression via an Autocrine Loop Orchestrated by EVs

Relevant findings suggest that EVs favor tumor progression via an autocrine loop in blood cancers, which includes interaction with their producing malignant cells, promoting survival and increasing aggressiveness. This mechanism of interplay is demonstrated in MM. MM cells and human MM cell lines release EVs that stimulate MM cell growth. Of interest, MM-derived EVs are enriched with CD147, a transmembrane molecule previously shown to be crucial for MM cell proliferation [87]. The same mechanism has been also described in CML. Specifically, LAMA84 CML cell-derived exosomes promote, through an autocrine mechanism, the proliferation and survival of tumor cells, both in vitro and in vivo, by the activation of an anti-apoptotic pathway regulated by exosome-associated TGF-β1 [88].

## 6. EV Regulation on Normal Hemopoiesis Restrain/Transformation

In blood cancers, the malignant clone occupies the bone marrow niche of the normal hemopoietic stem cells, restraining their survival, proliferation, and differentiation program. This results in the block of differentiation and proliferation of residual normal hemopoietic stem cells as well as in the disruption of the generation of normal blood cells, predisposing patients to anemia, hemorrhage, and infections.

### 6.1. Acute Myeloproliferative Disorders

There is increasing evidence of a leukemia-like phenotype induction in normal hemopoietic stem/progenitor cells by leukemia EVs. A recent study demonstrated the ability of EVs derived from leukemia cells (HL-60 and NB-4 cell lines) to induce proliferation of cord blood-derived CD34+ cells via miR-21/miR-29 dysregulation [89,90].

It has been also reported that AML cell-derived exosomes carrying miR-4532 have a suppressive effect on normal hemopoiesis via the activation of the signal transducer and activator of transcription 3 (STAT3) signaling pathway [91]. Interestingly, AML-EVs contribute to niche-dependent and reversible quiescence, but DNA damage persists in long-term residual normal hemopoietic stem cells. Mechanistically, to elicit long-term hemopoietic stem cell quiescence, AML-EVs transfer miRs, including miR-1246, that target the mTOR subunit Raptor, causing impairment of protein synthesis in long-term hemopoietic stem cells [92]. Accordingly, a prior study demonstrated that AML exosomes participate in the suppression of residual hematopoietic function either directly and indirectly via stromal components [93]. Another study revealed the presence of direct cross-talk between leukemic and residual hemopoietic cells in the bone marrow and that, in AML, the loss of normal hematopoietic function is in part a consequence of AML exosome-directed miR trafficking to hemopoietic stem progenitor cells. Consequently, exosomes isolated from cultured AML cells or the plasma mice bearing AML xenografts were miR-150 and miR-155-enriched. Hemopoietic stem progenitor cells co-cultured with either of these exosomes exhibited impaired clonogenic ability through the miR-150- and miR-155-mediated suppression of c-Myb, a transcription factor involved in hemopoietic stem cell differentiation and proliferation [94].

### 6.2. Chronic Myeloproliferative Disorders

Transferred tumor genes from EVs in vivo may represent an important mechanism for tumorigenesis. Based on a mouse model, it has been shown that the *BCR/ABL* hybrid gene can be transferred from EVs in vivo, resulting in CML. Specifically, the injection of K562 EVs in NOD/SCID mice causes de novo BCR/ABL mRNA and protein synthesis [95]. Consistently, Zhang et al. found that miR-146b-5p, which was highly expressed in EVs from the K562 CML cell line, coordinates the regulation of cancer-related genes to promote leukemic transformation. Notably, the treatment of mononuclear cells (from mobilized peripheral blood of healthy donors) with EVs from K562 cells expressing mimics of miR-146b-5p accelerates the transformation process mostly by silencing the tumor-suppressor NUMB [96].

Altogether, these data suggest that EVs from leukemic cells are involved in mediating two crucial processes for blood cancer development/maintenance: on one side, the ability to force normal cells toward a tumor phenotype and on the other side the inhibition of normal hemopoiesis. In particular, EV miR content seems to play an essential role in promoting leukemic transformation and/or inhibiting normal hemopoiesis (Supplementary Materials Table S4).

## 7. Angiogenesis Promotion Modulated by EVs

Angiogenesis has been shown to regulate the progression of blood cancers. In fact, EVs from blood cancer cells have been described to be key regulators in the maintenance and education of the bone marrow microenvironment by targeting not only stromal cells and immune cells but also vascular cells.

### 7.1. Acute Myeloproliferative Disorders

For instance, Acute Promyelocytic Leukemia-derived NB4 cells produce EVs with endothelial stimulating activity. Specifically, these EVs contain several PML–RARα (ATRA)-regulated vascular effector proteins and transcripts (Tissue Factor (TF), VEGF, IL-8). Importantly, PML–RARα modulate EV production and angiogenic cargo in acute promyelocytic leukemia cells [97]. Besides this, AML EVs enriched in pro-angiogenic factors (VEGF and VEGF receptor) can transfer them to endothelial cells, promoting vascular remodeling with the increase in endothelial cell glycolysis [98].

### 7.2. Chronic Myeloproliferative Disorders

The addition of EVs from LAMA84 CML cells to the human vascular endothelial cells (HUVEC) cell line increases survival and endothelial cell motility by promoting the expression of both ICAM-1 and VCAM-1 cell adhesion molecules and IL-8. Similarly, it has been shown that LAMA-84 CML cell-derived EVs are internalized by HUVEC cells during tubular differentiation, thereby promoting the process of neovascularization. Moreover, the transfer of CML (LAMA-84 cell line)-EV-miR-126 targets CXCL12 and vascular cell adhesion molecules in HUVEC, modulating the adhesion and migration of CML cells [99,100]. In particular, K562 CML cell-derived exosomes are internalized by endothelial cells and induce angiogenic activity in HUVEC cells. It has also been recorded that miR-92a enriched-EVs from K562 cells stimulate the migration and vascular tube formation of HUVEC [101]. Thus, EVs secreted by K562 CML cells can potentially influence in vitro and/or in vivo angiogenesis by stimulating angiotube formation through the activation of Src. Finally, CML-related therapy may influence exosome release/effects. Meanwhile, both imatinib and dasatinib reduce exosome release from K562 cells and only dasatinib blocks the exosome effect on endothelial cells [101,102]. Notably, endothelial cells acquire BCR-ABL RNA and the oncoprotein after incubation with EVs released from both K562 cells or the plasma of newly diagnosed CML patients [103].

Hypoxia plays an important role during the evolution of cancer cells. It has been found that the exosomes secreted from K562 CML cells in hypoxic conditions significantly enhance tube formation by HUVEC compared with exosomes produced in normoxic conditions. Notably, hypoxic exosomes from K562 CML cell lines show a distinct miR phenotype with higher levels of miR-210 [104].

### 7.3. Multiple Myeloma

It has been reported that MM exosomes, via their cargo of angiogenic proteins, promote endothelial cell growth, proliferation, and invasion [105]. Similar to CML, the bone marrow of MM patients becomes more hypoxic due to the overproduction of plasma cells, stimulating MM cells to produce higher amounts of exosomes compared to the normoxic conditions [106]. Consistently, Umezu et al. [107] described that hypoxic MM cells are enriched in miR-135b, which promotes in vitro angiogenesis. In further studies from this group, it has also been demonstrated that exosome miR-340 derived from young bone marrow stromal cells inhibit MM angiogenesis via the hepatocyte growth factor (HGF)/c-MET signaling pathway. Notably, the anti-angiogenic effect of exosomes from older bone marrow stromal cells was restored by direct transfection of young bone marrow stromal cell-derived exosomal miR, suggesting that exosomal miR replacement might have the therapeutic potential [108]. EVs from MM patients harbor CD138, which is an angiogenic regulator, and the angiogenic activity has been confirmed using mouse MM-derived EVs in vivo [105,109]. Finally, MM-derived EVs, enriched in the Piwi-interacting RNA-823, promote the proliferation, tube formation, and invasion of endothelial cells by enhancing the expression of VEGF, IL-6, and ICAM-1 and reducing apoptosis [110].

### 7.4. Lymphoma

EVs derived from lymphoma cells were shown to express c-Myc, Bcl-2, Mcl-1, CD19, and CD20 and stimulate angiogenesis by delivering angiogenic proteins, including VEGF [111]. Furthermore, it has been reported that exosomes-derived from adult T-cell leukemia/lymphoma cells regulate the properties of human MSC by transferring miR-21, miR-155, and VEGF, resulting in NF-κB activation and leading to the increased proliferation and expression of genes linked to migration and angiogenesis [112].

Altogether, these findings propose EVs as important mediators of angiogenesis in blood cancers, contributing to propagating angiogenic signals in the microenvironment and, ultimately, favoring tumor growth. This EV-driven activity is mainly referred to the ability of EVs in transferring miR and proangiogenic factors to endothelial cells (Supplementary Materials Table S5). Additionally, these results suggest a novel EV-based anticancer strategy aimed at blocking angiogenesis and consequently decreasing the growth of blood cancer cells.

## 8. The immune Evasion Mechanism of Blood Cancers: Focus on EVs

The ability of cancer cells to evade immune surveillance is a key mechanism for their development and maintenance. Recent reports support evidence that EVs from blood cancer patients contribute to the development of an immune suppressive microenvironment to create a “tumor friendly” niche. These EV effects are reported in various blood cancers, including AML [112], MM [103,111,112], and lymphomas [113].

### 8.1. Acute Myeloproliferative Disorders

Natural killer (NK) cells play a key role in immunosurveillance and Natural Killer Group 2, member D (NKG2D), is an NK cell receptor that, after activation, promotes the cytotoxic elimination of transformed cells. Growing evidence supports the notion that NK cell activity can be affected by EVs. Accordingly, a novel mechanism has been observed in AML where NK cell suppression is mediated by AML-derived EVs with the ability of IL-15 to counteract this suppression. Specifically, AML EVs decrease the NK cell cytotoxicity and down-regulate the expression of NKG2D in normal NK cells. Interestingly, neutralizing anti-TGF-β1 antibodies inhibits the EV-mediated suppression of NK cell activity and NKG2D down-regulation [22]. Consistently, NK cell co-incubation with AML exosomes carrying TGF-β1 induces the down-regulation of NKG2D expression [114].

### 8.2. Chronic Lympho/Myeloproliferative Disorders

It has been described that CML-derived EVs promote M2 macrophage polarization with TNF-α and IL10 over-expression [115].

Also, we recently demonstrated that circulating monocytes from JAK2 V617F-mutated MF patients are dysregulated and show a reduced in vitro ability to produce/secrete inflammatory cytokines in response to an infectious stimulus. Surprisingly, the JAK1/2 inhibitor Ruxolitinib promotes the EV-based inflammatory cytokine signaling [116].

In CLL, monocytes and macrophages are skewed toward protumorigenic phenotypes, with the release of tumor-promoting cytokines and the expression of immunosuppressive molecules such as programmed cell death 1 ligand 1 (PD-L1). Consistently, it has been demonstrated that the exosome-mediated transfer of a non-coding RNA (hY4) to monocytes contributes to cancer-related inflammation and immune escape via PD-L1 expression [117]. Furthermore, it is known that malignant cells support myeloid-derived suppressor cells’ (MDSC) inducing capacities [118]. Remarkably, under the effect of miR-155-enriched exosomes from CLL patients, monocytes differentiate into MDSC. Notably, this immune-regulatory interplay can be stopped by vitamin D, which negatively regulates miR-155 expression in CLL cells [119]. Reiners et al. found that transfer mRNA molecules by CLL EV to monocytes and fibroblasts spread tumor signals within the CLL microenvironment [120]. Clearly, CLL EVs are enriched in disease-relevant mRNA, including splicing factors, the TCL1A oncogene, and tyrosine kinases [121]. Additionally, they found that dysregulated balance of exosomal vs. soluble BCL-2-associated athanogene 6 (BAG-6) expression may favor immune evasion of CLL cells (115). Furthermore, the miR profiling of EVs released from CLL cells after stimulation with CD40 and IL4 has been characterized. They showed that EVs derived from CD40/IL4-stimulated CLL cells are enriched with miR, including miR-363. In addition, autologous CD4+ T cells that internalize CLL-EV containing miR-363 show increased migration, immunological signaling, and interactions with tumor cells, suggesting a role for CLL-EV in regulating T-cell function [122].

### 8.3. Multiple Myeloma

An intriguing fact reported in MM is that stromal cells from the bone marrow of patients release EVs in vitro which are uptaken by MDSC, supporting their survival through the activation of the STAT1/3 pathways and increasing the anti-apoptotic proteins Bcl-xL and Mcl-1. Furthermore, these exosomes increase the nitric oxide release from MM MDSC and enhance their suppressive activity on T cells, demonstrating that EVs derived from bone marrow stromal cells increase the immunosuppression that favors MM progression [123].

Conversely, EV-driven immune stimulatory activity has also been described in MM. According to AML EVs, it has been shown that exosomes derived from drug-induced senescent MM cells express IL15RA and stimulate NK cell proliferation in the presence of exogenous IL15. Thus, the exosome-mediated regulation enhances the NK cell-driven immune surveillance against cancer and provides new insights for the exploitation of senescence-based cancer therapy [124]. Furthermore, another group demonstrated that MM cells previously treated with sub-lethal doses of the alkylating agent melphalan are capable of releasing EVs with the potential to stimulate the production of IFN-γ by NK cells via the NF-KB pathway [125].

### 8.4. Lymphoma

Lymphoma cell-derived EVs that are enriched in NKG2D ligands also contribute to immune escape. Specifically, exosomes bearing the NKG2D ligand downregulate the in vitro NKG2D receptor-mediated cytotoxicity and, in turn, inhibit NK-cell function [113].

Several recent studies indicate that Epstein-Barr virus (EBV) can manipulate the local microenvironment by excreting viral and cellular components in EVs. Consistently, it has been observed that EBV-associated lymphomas secrete lymphoma cell-derived EVs that are mainly internalized into monocytes/macrophages and promote tumor evasion by inducing the immune regulatory phenotype in macrophages and enhancing the expression of TNF-α, IL-10, and arginase 1 [126]. Additionally, EBV hijacks the exosome pathway to excrete viral and cellular components that can modulate its microenvironment. EBV infection in B cells may induce T-cell inhibition via lymphoma cell-derived EV-mediated apoptosis [127]. Previous studies have shown also an immunosuppressive effect of exosomes from EBV-infected cells on recipient cells, such as human T cells and dendritic cells [128,129]. Furthermore, Gutzeit et al. demonstrated that the exosomes released from EBV-infected B cells can deliver their content to B cells and, thereby, stimulate B cell proliferation, the activation of induced cytidine deaminase, and class-switch recombination in B cells [130]. Moreover, recent data have shown that a network of EVs might participate in the tumor-supporting communication between malignant and proinflammatory immune cells in classical HL. EVs from HL cells express the CD30 receptor and can modify the microenvironment via interactions between their ligands (CD30L+) and neighboring cells. Specifically, bidirectional CD30-CD30L+ signals contribute to the education of distant immune cells, by stimulating their IL-8 release in a CD30-dependent mechanism [131]. It has also been documented that exosomes derived from Diffuse Large B Cell Lymphoma (DLBCL) cell lines (OCI-LY3, SU-DHL-16 (human DLBCL cell lines), and Raji cells (human Burkitt lymphoma cells) display malignancy molecules, such as c-Myc, Bcl-2, Mcl-1, CD19, and CD20, and mainly act as an immunosuppressive mediator, thereby promoting tumor growth in vivo. On other hand, it has been reported that DLBCL exosomes can also mediate and enhance dendritic cell-based antitumor immunity [111]. Recently, it has been described a further mechanism by which lymphoma B cell-derived EVs are involved in immune cell reprogramming. Specifically, exosomes derived from lymphoma B-cells and harboring mutated Myeloid Differentiation primary response 88 (MYD88) promote the activation of proinflammatory signaling in mast cells and macrophages, hence contributing to reprogramming the bone marrow microenvironment into a pro-tumorigenic niche [132].

To sum-up, EVs derived from blood cancer cells use different strategies to boost the immunosuppressive microenvironment and promote tumor growth. In particular, these EVs inhibit NK cell cytotoxicity, promote T cell and dendritic cell inhibition and M2 macrophage polarization, and enhance the immunosuppressive activity of MDSC in vitro and in vivo (Supplementary Materials Table S6). It is noteworthy that an EV-driven inflammatory microenvironment contributes to the growth and maintenance of the malignant clone(s). Critical aspects of this EV-orchestrated immunosuppression are the inability to distinguish the effects of malignant vs. normal cells-derived EVs since circulating EVs may derive from both compartments. Besides, EVs released from immune and non-immune cells play an important role in immune regulation. Therefore, altogether these data reinforce the importance of providing a deeper characterization of the origin of EVs, cargo and function in blood cancers to early define the EV-driven strategy of immune escape and potentially provide novel targeted therapeutic approaches.

## 9. EV Functions Related to the Hypercoagulable State of Blood Cancers

Thrombosis contributes to morbidity and mortality in blood cancers. The prevention of thrombotic events is thus a primary aim of the current treatment for these disorders. The genesis of thrombosis in these disorders is multifactorial and derived from a functional interplay among blood cells, endothelium and the coagulation system. Notably, the procoagulant role of circulating EVs in hematologic malignancies is increasingly acknowledged.

### 9.1. Acute Myeloproliferative Disorders

EVs may be predictors of thrombogenicity in patients with newly diagnosed AML. EV procoagulant activity has been reported to be elevated at diagnosis and in remission, and, unlike controls’ EVs, patients’ EVs increase endothelial cell thrombogenicity [23]. The role of EVs derived from acute promyelocytic leukemia cells has been assessed, as well as the contribution of Tissue Factor (TF) and phosphatidylserine (PS) of EVs to activate the coagulation cascade. Indeed, NB4 cells produce EVs and their procoagulant activity is related to the expression of TF and PS [133]. Additionally, EV procoagulant activity might be considered as a potential biomarker for the risk of thrombosis [134]. Recently, the effect of chemotherapy on pro-coagulant activity, PS exposure, and the TF activity of EVs derived from Jurkat cells (a human lymphoblastic leukemia cell line) have been tested. Interestingly, cells were treated with Vincristine (VCR) or Daunorubicin (DNR), at relevant concentrations, showing that VCR increased the procoagulant activity of Jurkat cells predominantly through PS exposure and increased EV release [135].

### 9.2. Chronic Myeloproliferative Disorders

Chronic myelomonocytic leukemia (CMML) is a myeloid hematological malignancy with overlapping characteristics of MDS and MPN. It has been reported that CMML monocyte EVs confer a procoagulant activity to healthy donor MSC, which can be reversed by an anti-TF antibody [136].

In MPN, elevated plasma levels of procoagulant EV represent a novel risk factor for thrombosis. It was firstly described by Duchemin et al. that, in polycythemia vera (PV) and essential thrombocythemia (ET) patients, the occurrence of an acquired “thrombomodulin-resistance” is partly due to circulating EVs. This thrombomodulin-resistance might contribute to the hypercoagulable state observed in MPN patients [137]. Consistently, PV patients are characterized by increased circulating procoagulant EVs with PS exposure, which could contribute to the hypercoagulable state in these patients [138]. An increased EV-related procoagulant activity has also been confirmed in patients with ET. Notably, the EV-related procoagulant activity was significantly greater in JAK2 V617F positive compared to unmutated patients and normal subjects. However, no difference was observed between the thrombosis and no thrombosis group [139].

Comparing ET, PV, and MF, increased circulating EV procoagulant activity was found in MPN patients, with the highest level in patients with PV as compared with both ET and MF patients. Remarkably, patients with a history of venous thrombosis have higher EV procoagulant activity. Furthermore, the presence of the JAK2 V617F mutation is associated with an increased procoagulant activity, as well as the higher JAK2 V617F variant allele frequency [140]. Consistently, in molecularly annotated ET patients at diagnosis, JAK2 V617F-mutated patients have more circulating EVs and higher levels of EVs with procoagulant activity than the Calreticulin-mutated and triple-negative counterparts. This could be partly explained by platelet activation, as assessed by P-selectin expression on EVs of the JAK2-mutated patients. Indeed, a relation between EV counts and the thrombotic risk was also observed. Therefore, circulating EVs might account, at least in part, for the distinct thrombotic risks according to mutational status in ET [141].

Additionally, Taniguchi et al. described that the plasma levels of procoagulant EVs expressing TF were higher in patients with thrombotic events than in patients without such events. Among patients who developed thrombosis, irrespective of the patients’ blood counts, TF+ EVs were increased in patients without cytoreductive therapy compared to those receiving cytoreductive therapy. These results suggest that increased circulating levels of TF+ EVs might be considered as a marker of thrombotic events in MPN patients [142].

### 9.3. Multiple Myeloma

It has been found that newly diagnosed MM patients have more and larger plasma EVs and that these EVs show procoagulant activity, resulting in an increased thrombin generation and TF and procoagulant phospholipids activity. This EV-mediated procoagulant effect diminishes after the initiation of high-dose induction therapy, suggesting that an EV-driven mechanism may contribute to the increased risk of thrombotic events in MM [143].

To summarize, these data highlight the relevance of circulating EVs in the thrombotic complications of patients with blood cancers (Supplementary Materials Table S7). Specifically, the expression of TF and PS on EVs seems to play a pivotal role in the activation of the hemostatic system. However, validated and reproducible results are still missing. Future research focusing on the screening of specific EV subsets based on their cargo and membrane composition/phenotype might help to identify biomarkers for hemostatic complications in patients with blood cancers.

## 10. Drug Resistance Shaped by EVs

The poor prognosis of blood cancers is due to multiple factors, including resistance to conventional and experimental therapies. Recent experimental findings support the role of EVs as key players in chemoresistance [2].

### 10.1. Acute Myeloproliferative Disorders

Leukemia stem cells are responsible for AML chemotherapy resistance and relapse. Growing evidence support the notion that EVs are involved in the mechanism of chemoresistance in AML. Of note, exosome-derived miR may participate in chemoresistant processes by regulating the expression of their target genes [144]. Bouvy et al. [145] focused on the role of EVs in the chemoresistance of AML. For this purpose, the drug-sensitive promyelocytic leukemia HL60 cell line and its multiresistant strain, HL60/AR, which overexpresses the multidrug resistance protein 1, were studied. A chemoresistance transfer between the two strains was obtained by treating HL60 cells with EVs generated by HL60/AR. In this context, two miRs (miR-19b and miR-20a) were differentially expressed in the EVs of sensitive vs. chemoresistant cells, suggesting a potential role of EVs as chemoresistance biomarkers in AML. Interestingly, another study demonstrated that reinitiating the senescence of leukemic stem cells via increased miR-34c-5p expression may be a new strategy for the treatment of AML patients. In detail, miR-34c-5p, a miR central to senescence network, was found to be significantly down-regulated in AML leukemic stem cells compared to in the normal counterparts. The lower expression of miR-34c-5p in the leukemic stem cells was related to adverse prognosis and chemoresistance. Interestingly, the miR-34c-5p deficiency in leukemic stem cells was due to the exosome-mediated transfer of miR-34c-5p. In fact, a forced increase in miR-34c-5p expression induced leukemic stem cell senescence ex vivo, prevented leukemia development and promoted the eradication of leukemic stem cells in immune-deficient mice [146].

Additionally, signals from the microenvironment also play a significant role. Bone marrow stromal cells in AML contribute to extrinsic drug resistance, generally through cell-cell contact or soluble cytokines. However, exosomes from AML bone marrow stromal cells have altered cytokine levels and miR cargo and confer chemoresistance to AML cells, suggesting that stromal exosome trafficking might be a candidate mechanism for chemoresistance in AML. In particular, stromal cells in AML release exosomes enriched for known clinical risk factors, including TGF-β1, miR155, and miR375 [147]. Consistently, exosomes from the MSC of AML patients have been reported to protect leukemic cells from treatment with the AC220 (specific fms such as tyrosine kinase [FLT]3 inhibitor) by carrying the FLT3 internal tandem duplication [148]. Another study conducted by Wang et al. demonstrated that bone marrow stromal cells-derived exosomes protect human B-cell acute lymphoblastic leukemia cells from etoposide-induced apoptosis by inducing exosome-driven drug resistance [149].

### 10.2. Chronic Myeloproliferative Disorders

Further experimental evidence showed that exosomes derived from imatinib-resistant CML cells can be internalized into sensitive CML cells and induce drug-resistance by delivering miR365. This is principally due to the fact that the exosomal transfer of miR365 induces drug resistance by inhibiting the expression of pro-apoptosis proteins in sensitive CML cells [150]. Furthermore, it is known that ABC transporters, such as P-glycoprotein (P-gp), play a key role in multidrug resistance. Multidrug-resistant cancer cell lines (including the chronic myeloid leukemia cell line K562) overexpressing P-gp acquire a different metabolic profile from their drug-sensitive counterpart cells, and the EVs released by multidrug-resistant cells induce a metabolic switch towards the multidrug-resistant phenotype in the recipient cells. These include (i) alterations in the glutathione metabolism, (ii) alterations in epigenetic regulation, (iii) increased rates of glycolysis, and (iv) changing the phenotype of the surrounding drug-sensitive cells by EV-mediated transfer. In conclusion, these results highlight the role of EVs in the intercellular transfer of multidrug resistance in CML and suggest that specific metabolic alterations may represent a target for overcoming multidrug resistance [151].

### 10.3. Multiple Myeloma

Regarding MM, Krishnan et al. recently demonstrated that circulating EVs can be used to monitor disease burden, disease progression, and the development of chemoresistance. These EVs are characterized by the expression of the following biomarkers: plasma-cell marker (CD138), multidrug resistance protein (P-gp), stem-cell marker (CD34), and PS. Elevated levels of P-gp+ and PS+ EVs correlate with disease progression and chemoresistance [152]. It is noteworthy that a prior study corroborated that serum exosomal miR may be used as drug resistance biomarkers for MM. Specifically, the microarray profiling found that the expression of four exosomal miRs (miR16, miR15a, miR20a, and miR17) was down-regulated in patients resistant to Bortezomib [153]. Once again, the bone marrow niche might play a relevant role. It has been described that bone marrow stromal cells-derived exosomes induce cell survival and the chemoresistance of MM cells to bortezomib through the activation of survival pathways, including c-Jun N-terminal kinase, p38, p53, and Akt [153,154]. Additionally, lncRNAs derived from MSC exosomes are involved in the communication between MSC and MM cells, thus resulting in proteasome inhibitors resistance in MM cells [155]. Notably, it has been shown that conventional chemotherapy including melphalan and bortezomib can promote exosome production by MM cells, especially those that survive chemotherapy. These exosomes are enriched in surface heparanase enzyme, which is involved in chemoresistance. After exosome uptake, MM cells show the activation of the ERK pathway via the delivery of their heparanase content, the induction of TNF-α production by macrophages, matrix degradation, and migration promotion [156].

### 10.4. Lymphoma

The relevance of EVs as predictors of drug response has also been demonstrated in patients with lymphoma. Feng et al. found increased levels of miR99a and miR-125b in exosomes derived from DLBCL patients’ serum. Notably, exosomal miR levels were correlated with shorter progression-free survival and chemoresistance [157]. Anti-CD20-based therapy has been developed and approved for lymphoma patients. However, it has been suggested that since B-cell lymphoma cells release CD20+ lymphoma cell-derived EVs, these CD20+ EVs can capture rituximab and thus constrain its therapeutic effectiveness. This seems to be particularly relevant at the beginning of treatment [158]. The export of cytostatic drugs has been recognized to contribute to tumor cell drug resistance. Indeed, it has been reported that lymphoma cells extrude anthracyclines in exosomes, that the biogenesis of exosomes can be suppressed by silencing the ABC transporter A3, and that this pathway is targetable by the cyclooxygenase inhibitor indomethacin. Therefore, restraining exosome biogenesis might be a promising approach to overcome drug resistance [159].

The expression of HSP-70, c-Myc, Bcl-2, Mcl-1, xIAP, and Bcl-xL, as well as other molecules, such as phosphatidylinositol, ERK, MAPK, chemokines, cell surface receptors, and G proteins in lymphoma cell-derived EVs, has also been linked to resistance to immunotherapy [111]. Interestingly, the increase in ADAM (A Disintegrin And Metalloproteinase)10 activity, which is mediated by lymphoma cell-derived EV, has been reported to interfere with immunotherapeutic approaches. The release of TNF-α, soluble MHC I polypeptide-related sequence A (sMICA), and soluble CD30 has been described as a pivotal mechanism. This observation has led to specific ADAM10 inhibitors being proposed to favor the anti-lymphoma immune response and/or drive efficient immunotherapy [160].

These findings reveal the profound effects of EVs, both blood cancer cell-derived and microenvironment-derived, on modifying malignant cells in the development of drug resistance (Supplementary Materials Table S8). In addition, since intratumoral heterogeneity is a major determinant in developing resistance that underpins treatment failure, EVs have the potential to detect heterogeneity and enhance our ability in the longitudinal monitoring of the dynamic clonal evolution of tumor cells during chemotherapy. Therefore, future studies and controlled trials might focus on patient EV characterization at diagnosis and during follow-up in order to further understand the EV-driven “smart strategy” of chemoresistance in blood cancers and facilitate the early detection of drug resistance.

## 11. EVs as a Drug Delivery System

Growing evidence shows that EVs can be used as a “smart” drug delivery system in cancer, promoting target specificity and reducing off-target side effects. This is principally due to the ability of EVs to retain stable concentrations of the loaded molecules. Two options can be pursued. In the first one, due to the ability of cells to encapsulate exogenous molecules and release them as vesicles, EVs released by drug-treated cells can be used to deliver chemotherapeutic agents to tumor cells. Conversely, the second one is based on the fact that EVs can encapsulate drugs and deliver them to the target cells for therapeutic purposes [161]. To date, no EV-based drug delivery strategy has been described for hematological malignancies. Only Bellavia D et al. recently demonstrated that IL-3-targeted exosomes can deliver Imatinib to CML cells in order to overcome pharmacological resistance [162].

Several clinical trials involving EV are currently ongoing; however, they refer to solid tumors. Notably, only the ExoReBly project (NCT03985696) aims to characterize EVs from lymphoma patients and use them as a marker of response to therapy and disease outcome.

Taken together, these data depict a situation that is at its infancy in hematologic malignancies. Hopefully, more applications of EVs as drug delivery tools for therapeutic purposes will arise soon.

## 12. Conclusions

**Figure 2:**
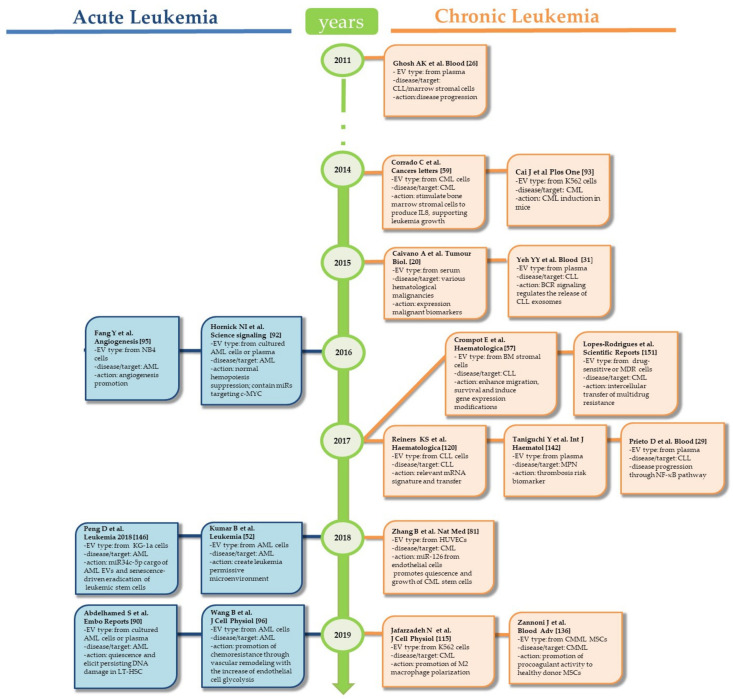
Timeline of key discoveries in EVs from leukemia.

**Figure 3:**
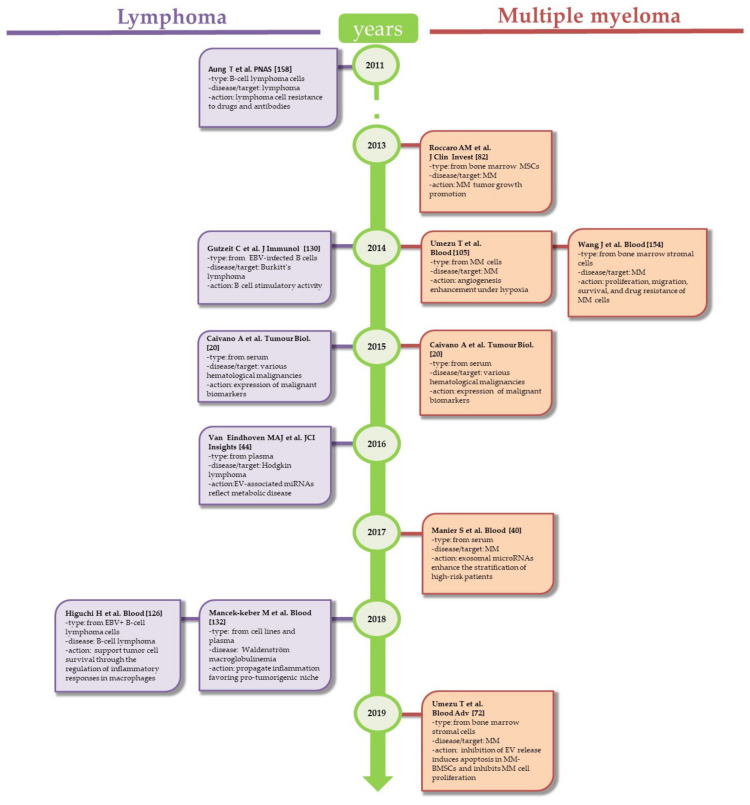
Timeline of key discoveries in EVs from Lymphoma and Multiple Myeloma.

Here, we analyzed the current literature specifically related to the EV “smart intelligence strategy” of blood cancers (Figure 2 and Figure 3). Since microenvironmental signals are involved in the development and progression of blood cancers, new ways to detect them through EVs would be highly valuable for diagnosing and monitoring hematological malignancies. In particular, this new form of intercellular communication appears to effectively orchestrate many biological events related to the development, maintenance, and progression of malignant cells. In fact, accumulating evidence from preclinical and clinical studies demonstrates that EVs released from blood cancer cells and/or (bone marrow) the microenvironment are involved in the pathogenesis of these disorders. Most of the information points to an important role of EVs in reinforcing the functional behavior of blood cancer cells and their interaction with the microenvironment (bone marrow), leading to escape from immune surveillance and promoting a thrombotic state. The emerging consequence of this EV-driven complex interaction may result in the development of chemoresistance.

## 13. Future Prospects

Despite the complexity, identifying the original cell type that releases EVs might be crucial in the creation of a clinically relevant database of chart types, phenotypes, properties, and cargo of EVs from various hematological malignancies. Also, taking into account the different sources of EVs (e.g., plasma, serum, urine), it is also essential to gain a more comprehensive understanding of how EV profiling is associated with disease burden and evolution.

Achieving a deeper knowledge of this intricate communication system would allow us to identify its weaknesses. Indeed, a better knowledge of the mechanisms promoted and/or regulated by EVs concerning the bone marrow niche and the immune system is of particular relevance in the context of developing new drugs for the personalized therapy of blood cancer patients. In addition, the future prospects of EVs in blood cancers may include their role as a biological tool for the detection/monitoring of minimal residual disease and also mutational tracking during therapy to measure the onset of chemoresistance, with potential implications in the treatment decision.

Notably, despite the acknowledged role of EVs as blood cancer biomarkers, the applicability of EVs for tailoring therapy decisions or monitoring disease progression is still far away. This is mainly due to the fact that the basic mechanisms/characteristics of EV biology in blood cancers have yet to be fully determined. Currently, the huge debate on EV compositions and protocols for EV isolation and characterization reveals several issues that still need to be explored.

Therefore, continued in-depth investigation is required. Ultimately, having a deep understanding of EVs will provide a better clinical translational potential for their use in blood cancers.

## Data Availability

No new data were created or analyzed in this study. Data sharing is not applicable to this article.

## Supplementary Materials

The following are available online at https://www.mdpi.com/2073-4425/12/3/416/s1: Table S1: Summary of current studies on EVs as biomarkers, Table S2: Summary of current studies on EVs: re-education of the bone marrow niche, Table S3: Summary of current studies on EVs: signals from microenvironment, Table S4: Summary of current studies on EVs: normal hemopoiesis restrain/transformation, Table S5: Summary of current studies on EVs: angiogenesis promotion, Table S6: Summary of current studies on EVs: immune evasion, Table S7: Summary of current studies on EVs: hypercoagulability, Table S8: Summary of current studies on EVs: drug resistance..

## Abbreviations

ADAM	A Disintegrin And Metalloproteinase
ANGPTL	Angiopoietin Like
AML	Acute Myeloid Leukemia
ATRA	All trans retinoic acid
BAG-6	BCL-2-associated athanogene 6
BCL	B-cell lymphoma
BCR	B Cell Receptor
BCR-ABL	Break point cluster region/Abelson
BMSC	Bone marrow stromal cell
CLL	Chronic Lymphoid Leukemia
CML	Chronic Myeloid Leukemia
CMML	Chronic Myelomonocytic Leukemia
CXCL	C-X-C motif chemokine ligand
CXCR4	C-X-C chemokine receptor type 4
DLBCL	Diffuse Large B Cell Lymphoma
DNR	Daunorubicin
EBV	Epstein-Barr virus
ERK	Extracellular signal-regulated kinase
ET	Essential Thrombocythemia
EVs	Extracellular Vesicles
FGF	Fibroblast Growth Factor
FGFR	Fibroblast Growth Factor Receptor
FLT3	Fms-like tyrosine kinase 3
HSP	Heat shock protein
HL	Hodgkin Lymphoma
HGF	Hepatocyte Growth Factor
HSP-70	Heat shock Protein-70
HSC	Hemopoietic Stem Cells
HUVEC	Human umbilical vein endothelial cell
ICAM	Intercellular adhesion molecule-1
Ig	Immunoglobulin
IGF	Insulin Growth Factor
IL	Interleukin
IP-10	Interferon-γ-inducible protein 10
JAK	Janus Kinase
KO	Knock out
MAPK	Mitogen-activated protein kinase
MCP-1	Monocyte chemoattractant protein-1
MICA/MICB	MHC I polypeptide-related sequence A/B
MDS	Myelodysplastic Syndrome
MDSC	Myeloid-derived suppressor cell
MF	Myelofibrosis
miR	microRNA
MYD88	Myeloid differentiation primary response 88
MM	Multiple Myeloma
MPN	Myeloproliferative Neoplasms
MSC	Mesenchymal Stromal Cells
NOD/SCID	Humanized non-obese diabetic/severe combined immunodeficiency/interleukin-2 recep-tor-γ-null
NK	Natural Killer
NKG2D	Natural killer Group2, member D
PD-L1	Programmed Cell Death 1 ligand 1
P-gp	P-glycoprotein
PML-RARα	Promyelocytic leukemia gene-retinoic acid receptor α
PRINS	Psoriasis Susceptibility-related RNA Gene induced by stress
PS	Phosphatidylserine
PV	Polycythemia Vera
STAT	Signal transducer and activator of transcription
SDF	Stromal-derived factor
SMM	Smouldering Myeloma
SPRED1	Sprouty-related, EVH1 domain-containing protein 1
TF	Tissue Factor
TGF	Transforming Growth Factor
TKI	Tyrosine kinase inhibitor
TNF	Tumor Necrosis Factor
TPO	Thrombopoietin
VCAM	Vascular cell adhesion molecule
VCR	Vincristine
VEGF	Vascular endothelial growth factor
VPS33B	Vacuolar protein sorting protein 33b
WM	Waldenstrom Macroglobulinemia

## References

[1] Trino S., Lamorte D., Caivano A., De Luca L., Sgambato A., Laurenzana I. (2020). Clinical relevance of extracellular vesicles in hematological neoplasms: From liquid biopsy to cell biopsy. Leukemia.

[2] Longjohn M.N., Hudson J.B.J., Smith N.C., Rise M.L., Moorehead P.C., Christian S.L. (2020). Deciphering the messages carried by extracellular vesicles in hematological malignancies. Blood Rev..

[3] Gargiulo E., Paggetti J., Moussay E. (2019). Hematological Malignancy-Derived Small Extracellular Vesicles and Tumor Microenvironment: The Art of Turning Foes into Friends. Cells.

[4] Litwinska Z., Luczkowska K., Machalinski B. (2019). Extracellular vesicles in hematological malignancies. Leuk. Lymphoma.

[5] Ball S., Nugent K. (2018). Microparticles in Hematological Malignancies: Role in Coagulopathy and Tumor Pathogenesis. Am. J. Med. Sci..

[6] Ohyashiki J.H., Umezu T., Ohyashiki K. (2018). Extracellular vesicle-mediated cell-cell communication in haematological neoplasms. Philos. Trans. R. Soc. Lond. B Biol. Sci..

[7] Caivano A., La Rocca F., Laurenzana I., Trino S., De Luca L., Lamorte D., Del Vecchio L., Musto P. (2017). Extracellular Vesicles in Hematological Malignancies: From Biology to Therapy. Int. J. Mol. Sci..

[8] Khalife J., Sanchez J.F., Pichiorri F. (2020). Extracellular Vesicles in Hematological Malignancies: From Biomarkers to Therapeutic Tools. Diagnostics.

[9] Van Niel G., D’Angelo G., Raposo G. (2018). Shedding light on the cell biology of extracellular vesicles. Nat. Rev. Mol. Cell. Biol..

[10] Mathivanan S., Fahner C.J., Reid G.E., Simpson R.J. (2012). ExoCarta 2012: Database of exosomal proteins, RNA and lipids. Nucleic Acids Res..

[11] Pathan M., Fonseka P., Chitti S.V., Kang T., Sanwlani R., Van Deun J., Hendrix A., Mathivanan S. (2019). Vesiclepedia 2019: A compendium of RNA, proteins, lipids and metabolites in extracellular vesicles. Nucleic Acids Res..

[12] Kalra H., Simpson R.J., Ji H., Aikawa E., Altevogt P., Askenase P., Bond V.C., Borras F.E., Breakefield X., Budnik V. (2012). Vesiclepedia: A compendium for extracellular vesicles with continuous community annotation. PLoS Biol..

[13] Russell A.E., Sneider A., Witwer K.W., Bergese P., Bhattacharyya S.N., Cocks A., Cocucci E., Erdbrugger U., Falcon-Perez J.M., Freeman D.W. (2019). Biological membranes in EV biogenesis, stability, uptake, and cargo transfer: An ISEV position paper arising from the ISEV membranes and EVs workshop. J. Extracell Vesicles.

[14] Kalluri R., LeBleu V.S. (2020). The biology, function, and biomedical applications of exosomes. Science.

[15] Li Q., Wang H., Peng H., Huyan T., Cacalano N.A. (2019). Exosomes: Versatile Nano Mediators of Immune Regulation. Cancers.

[16] Chen Z., Larregina A.T., Morelli A.E. (2019). Impact of extracellular vesicles on innate immunity. Curr. Opin. Organ. Transplant.

[17] Othman N., Jamal R., Abu N. (2019). Cancer-Derived Exosomes as Effectors of Key Inflammation-Related Players. Front. Immunol..

[18] Wang T., Nasser M.I., Shen J., Qu S., He Q., Zhao M. (2019). Functions of Exosomes in the Triangular Relationship between the Tumor, Inflammation, and Immunity in the Tumor Microenvironment. J. Immunol. Res..

[19] Sharifi H., Shafiee A., Molavi G., Razi E., Mousavi N., Sarvizadeh M., Taghizadeh M. (2019). Leukemia-derived exosomes: Bringing oncogenic signals to blood cells. J. Cell Biochem..

[20] Chulpanova D.S., Kitaeva K.V., James V., Rizvanov A.A., Solovyeva V.V. (2018). Therapeutic Prospects of Extracellular Vesicles in Cancer Treatment. Front. Immunol..

[21] Caivano A., Laurenzana I., De Luca L., La Rocca F., Simeon V., Trino S., D’Auria F., Traficante A., Maietti M., Izzo T. (2015). High serum levels of extracellular vesicles expressing malignancy-related markers are released in patients with various types of hematological neoplastic disorders. Tumour Biol..

[22] Szczepanski M.J., Szajnik M., Welsh A., Whiteside T.L., Boyiadzis M. (2011). Blast-derived microvesicles in sera from patients with acute myeloid leukemia suppress natural killer cell function via membrane-associated transforming growth factor-beta1. Haematologica.

[23] Tzoran I., Rebibo-Sabbah A., Brenner B., Aharon A. (2015). Disease dynamics in patients with acute myeloid leukemia: New biomarkers. Exp. Hematol..

[24] Hornick N.I., Huan J., Doron B., Goloviznina N.A., Lapidus J., Chang B.H., Kurre P. (2015). Serum Exosome MicroRNA as a Minimally-Invasive Early Biomarker of AML. Sci. Rep..

[25] Jiang L., Deng T., Wang D., Xiao Y. (2018). Elevated Serum Exosomal miR-125b Level as a Potential Marker for Poor Prognosis in Intermediate-Risk Acute Myeloid Leukemia. Acta Haematol..

[26] Fang Z., Wang X., Wu J., Xiao R., Liu J. (2020). High serum extracellular vesicle miR-10b expression predicts poor prognosis in patients with acute myeloid leukemia. Cancer Biomark..

[27] Ghosh A.K., Secreto C.R., Knox T.R., Ding W., Mukhopadhyay D., Kay N.E. (2010). Circulating microvesicles in B-cell chronic lymphocytic leukemia can stimulate marrow stromal cells: Implications for disease progression. Blood.

[28] De Luca L., D’Arena G., Simeon V., Trino S., Laurenzana I., Caivano A., La Rocca F., Villani O., Mansueto G., Deaglio S. (2017). Characterization and prognostic relevance of circulating microvesicles in chronic lymphocytic leukemia. Leuk. Lymphoma.

[29] Belov L., Matic K.J., Hallal S., Best O.G., Mulligan S.P., Christopherson R.I. (2016). Extensive surface protein profiles of extracellular vesicles from cancer cells may provide diagnostic signatures from blood samples. J. Extracell Vesicles.

[30] Prieto D., Sotelo N., Seija N., Sernbo S., Abreu C., Duran R., Gil M., Sicco E., Irigoin V., Oliver C. (2017). S100-A9 protein in exosomes from chronic lymphocytic leukemia cells promotes NF-kappaB activity during disease progression. Blood.

[31] Boysen J., Nelson M., Magzoub G., Maiti G.P., Sinha S., Goswami M., Vesely S.K., Shanafelt T.D., Kay N.E., Ghosh A.K. (2017). Dynamics of microvesicle generation in B-cell chronic lymphocytic leukemia: Implication in disease progression. Leukemia.

[32] Yeh Y.Y., Ozer H.G., Lehman A.M., Maddocks K., Yu L., Johnson A.J., Byrd J.C. (2015). Characterization of CLL exosomes reveals a distinct microRNA signature and enhanced secretion by activation of BCR signaling. Blood.

[33] Farahani M., Rubbi C., Liu L., Slupsky J.R., Kalakonda N. (2015). CLL Exosomes Modulate the Transcriptome and Behaviour of Recipient Stromal Cells and Are Selectively Enriched in miR-202-3p. PLoS ONE.

[34] Wu Z., Sun H., Wang C., Liu W., Liu M., Zhu Y., Xu W., Jin H., Li J. (2020). Mitochondrial Genome-Derived circRNA mc-COX2 Functions as an Oncogene in Chronic Lymphocytic Leukemia. Mol. Ther. Nucleic Acids.

[35] Barone M., Ricci F., Sollazzo D., Ottaviani E., Romano M., Auteri G., Bartoletti D., Reggiani M.L.B., Vianelli N., Tazzari P.L. (2019). Circulating megakaryocyte and platelet microvesicles correlate with response to ruxolitinib and distinct disease severity in patients with myelofibrosis. Br. J. Haematol.

[36] Forte D., Barone M., Morsiani C., Simonetti G., Fabbri F., Bruno S., Bandini E., Sollazzo D., Collura S., Deregibus M.C. (2021). Distinct profile of CD34(+) cells and plasma-derived extracellular vesicles from triple-negative patients with Myelofibrosis reveals potential markers of aggressive disease. J. Exp. Clin. Cancer Res..

[37] Kim D.K., Cho Y.E., Komarow H.D., Bandara G., Song B.J., Olivera A., Metcalfe D.D. (2018). Mastocytosis-derived extracellular vesicles exhibit a mast cell signature, transfer KIT to stellate cells, and promote their activation. Proc. Natl. Acad. Sci. USA.

[38] Zhang J., Zhao A., Sun L., Chen W., Zhang H., Chen Z., Liu F. (2017). Selective surface marker and miRNA profiles of CD34(+) blast-derived microvesicles in chronic myelogenous leukemia. Oncol. Lett..

[39] Harshman S.W., Canella A., Ciarlariello P.D., Agarwal K., Branson O.E., Rocci A., Cordero H., Phelps M.A., Hade E.M., Dubovsky J.A. (2016). Proteomic characterization of circulating extracellular vesicles identifies novel serum myeloma associated markers. J. Proteom..

[40] Krishnan S.R., Luk F., Brown R.D., Suen H., Kwan Y., Bebawy M. (2016). Isolation of Human CD138(+) Microparticles from the Plasma of Patients with Multiple Myeloma. Neoplasia.

[41] Zhang Z.Y., Li Y.C., Geng C.Y., Zhou H.X., Gao W., Chen W.M. (2019). Serum exosomal microRNAs as novel biomarkers for multiple myeloma. Hematol. Oncol..

[42] Manier S., Liu C.J., Avet-Loiseau H., Park J., Shi J., Campigotto F., Salem K.Z., Huynh D., Glavey S.V., Rivotto B. (2017). Prognostic role of circulating exosomal miRNAs in multiple myeloma. Blood.

[43] Sedlarikova L., Bollova B., Radova L., Brozova L., Jarkovsky J., Almasi M., Penka M., Kuglik P., Sandecka V., Stork M. (2018). Circulating exosomal long noncoding RNA PRINS-First findings in monoclonal gammopathies. Hematol. Oncol..

[44] Yao Y., Wei W., Sun J., Chen L., Deng X., Ma L., Hao S. (2015). Proteomic analysis of exosomes derived from human lymphoma cells. Eur. J. Med. Res..

[45] Domnikova N.P., Dolgikh T.Y., Sholenberg E.V., Vorontsova E.V., Goreva O.B., Mel’nikova E.V., Gorbachenko E.A., Grishanova A.Y. (2013). Blood microvesicles during chronic lymphoproliferative diseases. Bull. Exp. Biol. Med..

[46] Van Eijndhoven M.A., Zijlstra J.M., Groenewegen N.J., Drees E.E., van Niele S., Baglio S.R., Koppers-Lalic D., van der Voorn H., Libregts S.F., Wauben M.H. (2016). Plasma vesicle miRNAs for therapy response monitoring in Hodgkin lymphoma patients. JCI Insight.

[47] Provencio M., Rodriguez M., Cantos B., Sabin P., Quero C., Garcia-Arroyo F.R., Rueda A., Maximiano C., Rodriguez-Abreu D., Sanchez A. (2017). mRNA in exosomas as a liquid biopsy in non-Hodgkin Lymphoma: A multicentric study by the Spanish Lymphoma Oncology Group. Oncotarget.

[48] Hurwitz S.N., Jung S.K., Kurre P. (2020). Hematopoietic stem and progenitor cell signaling in the niche. Leukemia.

[49] Batsali A.K., Georgopoulou A., Mavroudi I., Matheakakis A., Pontikoglou C.G., Papadaki H.A. (2020). The Role of Bone Marrow Mesenchymal Stem Cell Derived Extracellular Vesicles (MSC-EVs) in Normal and Abnormal Hematopoiesis and Their Therapeutic Potential. J. Clin. Med..

[50] Borgovan T., Crawford L., Nwizu C., Quesenberry P. (2019). Stem cells and extracellular vesicles: Biological regulators of physiology and disease. Am. J. Physiol. Cell Physiol..

[51] Houshmand M., Blanco T.M., Circosta P., Yazdi N., Kazemi A., Saglio G., Zarif M.N. (2019). Bone marrow microenvironment: The guardian of leukemia stem cells. World J. Stem Cells.

[52] Laurenzana I., Lamorte D., Trino S., De Luca L., Ambrosino C., Zoppoli P., Ruggieri V., Del Vecchio L., Musto P., Caivano A. (2018). Extracellular Vesicles: A New Prospective in Crosstalk between Microenvironment and Stem Cells in Hematological Malignancies. Stem Cells Int..

[53] Pando A., Reagan J.L., Quesenberry P., Fast L.D. (2018). Extracellular vesicles in leukemia. Leuk. Res..

[54] Kumar B., Garcia M., Weng L., Jung X., Murakami J.L., Hu X., McDonald T., Lin A., Kumar A.R., DiGiusto D.L. (2018). Acute myeloid leukemia transforms the bone marrow niche into a leukemia-permissive microenvironment through exosome secretion. Leukemia.

[55] Horiguchi H., Kobune M., Kikuchi S., Jomen W., Murase K., Ibata S., Iyama S., Sato T., Kamihara Y., Miyanishi K. (2014). Exosomes Derived from AML/MDS Cells Is Involved in Stromal Dysfunction and Bone Marrow Failure. Blood.

[56] Huan J., Hornick N.I., Shurtleff M.J., Skinner A.M., Goloviznina N.A., Roberts C.T., Kurre P. (2013). RNA trafficking by acute myelogenous leukemia exosomes. Cancer Res..

[57] Horiguchi H., Kobune M., Kikuchi S., Yoshida M., Murata M., Murase K., Iyama S., Takada K., Sato T., Ono K. (2016). Extracellular vesicle miR-7977 is involved in hematopoietic dysfunction of mesenchymal stromal cells via poly(rC) binding protein 1 reduction in myeloid neoplasms. Haematologica.

[58] Gu H., Chen C., Hao X., Wang C., Zhang X., Li Z., Shao H., Zeng H., Yu Z., Xie L. (2016). Sorting protein VPS33B regulates exosomal autocrine signaling to mediate hematopoiesis and leukemogenesis. J. Clin. Investig..

[59] Crompot E., Van Damme M., Pieters K., Vermeersch M., Perez-Morga D., Mineur P., Maerevoet M., Meuleman N., Bron D., Lagneaux L. (2017). Extracellular vesicles of bone marrow stromal cells rescue chronic lymphocytic leukemia B cells from apoptosis, enhance their migration and induce gene expression modifications. Haematologica.

[60] Corrado C., Raimondo S., Saieva L., Flugy A.M., De Leo G., Alessandro R. (2014). Exosome-mediated crosstalk between chronic myelogenous leukemia cells and human bone marrow stromal cells triggers an interleukin 8-dependent survival of leukemia cells. Cancer Lett..

[61] Paggetti J., Haderk F., Seiffert M., Janji B., Distler U., Ammerlaan W., Kim Y.J., Adam J., Lichter P., Solary E. (2015). Exosomes released by chronic lymphocytic leukemia cells induce the transition of stromal cells into cancer-associated fibroblasts. Blood.

[62] Corrado C., Saieva L., Raimondo S., Santoro A., De Leo G., Alessandro R. (2016). Chronic myelogenous leukaemia exosomes modulate bone marrow microenvironment through activation of epidermal growth factor receptor. J. Cell Mol. Med..

[63] Fu F.F., Zhu X.J., Wang H.X., Zhang L.M., Yuan G.L., Chen Z.C., Li Q.B. (2017). BCR-ABL1-positive microvesicles malignantly transform human bone marrow mesenchymal stem cells in vitro. Acta Pharmacol. Sin..

[64] Jiang Y.H., Liu J., Lin J., Li S.Q., Xu Y.M., Min Q.H., Zhong Q.H., Sun F., Li J., You X.H. (2020). K562 cell-derived exosomes suppress the adhesive function of bone marrow mesenchymal stem cells via delivery of miR-711. Biochem. Biophys. Res. Commun..

[65] Gao X., Wan Z., Wei M., Dong Y., Zhao Y., Chen X., Li Z., Qin W., Yang G., Liu L. (2019). Chronic myelogenous leukemia cells remodel the bone marrow niche via exosome-mediated transfer of miR-320. Theranostics.

[66] Raab M.S., Podar K., Breitkreutz I., Richardson P.G., Anderson K.C. (2009). Multiple myeloma. Lancet.

[67] Mitsiades C.S., Mitsiades N.S., Munshi N.C., Richardson P.G., Anderson K.C. (2006). The role of the bone microenvironment in the pathophysiology and therapeutic management of multiple myeloma: Interplay of growth factors, their receptors and stromal interactions. Eur. J. Cancer.

[68] Akhtar S., Ali T.A., Faiyaz A., Khan O.S., Raza S.S., Kulinski M., Omri H.E., Bhat A.A., Uddin S. (2020). Cytokine-Mediated Dysregulation of Signaling Pathways in the Pathogenesis of Multiple Myeloma. Int. J. Mol. Sci..

[69] Raimondi L., De Luca A., Amodio N., Manno M., Raccosta S., Taverna S., Bellavia D., Naselli F., Fontana S., Schillaci O. (2015). Involvement of multiple myeloma cell-derived exosomes in osteoclast differentiation. Oncotarget.

[70] Liu Z., Liu H., Li Y., Shao Q., Chen J., Song J., Fu R. (2020). Multiple myeloma-derived exosomes inhibit osteoblastic differentiation and improve IL-6 secretion of BMSCs from multiple myeloma. J. Investig. Med..

[71] Faict S., Muller J., De Veirman K., De Bruyne E., Maes K., Vrancken L., Heusschen R., De Raeve H., Schots R., Vanderkerken K. (2018). Exosomes play a role in multiple myeloma bone disease and tumor development by targeting osteoclasts and osteoblasts. Blood Cancer J..

[72] Raimondo S., Saieva L., Vicario E., Pucci M., Toscani D., Manno M., Raccosta S., Giuliani N., Alessandro R. (2019). Multiple myeloma-derived exosomes are enriched of amphiregulin (AREG) and activate the epidermal growth factor pathway in the bone microenvironment leading to osteoclastogenesis. J. Hematol. Oncol..

[73] Li B., Xu H., Han H., Song S., Zhang X., Ouyang L., Qian C., Hong Y., Qiu Y., Zhou W. (2018). Exosome-mediated transfer of lncRUNX2-AS1 from multiple myeloma cells to MSCs contributes to osteogenesis. Oncogene.

[74] Umezu T., Imanishi S., Yoshizawa S., Kawana C., Ohyashiki J.H., Ohyashiki K. (2019). Induction of multiple myeloma bone marrow stromal cell apoptosis by inhibiting extracellular vesicle miR-10a secretion. Blood Adv..

[75] De Veirman K., Wang J., Xu S., Leleu X., Himpe E., Maes K., De Bruyne E., Van Valckenborgh E., Vanderkerken K., Menu E. (2016). Induction of miR-146a by multiple myeloma cells in mesenchymal stromal cells stimulates their pro-tumoral activity. Cancer Lett..

[76] Cheng Q., Li X., Liu J., Ye Q., Chen Y., Tan S. (2017). Multiple Myeloma-Derived Exosomes Regulate the Functions of Mesenchymal Stem Cells Partially via Modulating miR-21 and miR-146a. Stem Cells Int..

[77] Frassanito M.A., Desantis V., Di Marzo L., Craparotta I., Beltrame L., Marchini S., Annese T., Visino F., Arciuli M., Saltarella I. (2019). Bone marrow fibroblasts overexpress miR-27b and miR-214 in step with multiple myeloma progression, dependent on tumour cell-derived exosomes. J. Pathol..

[78] Dorsam B., Bosl T., Reiners K.S., Barnert S., Schubert R., Shatnyeva O., Zigrino P., Engert A., Hansen H.P., von Strandmann E.P. (2018). Hodgkin Lymphoma-Derived Extracellular Vesicles Change the Secretome of Fibroblasts Toward a CAF Phenotype. Front. Immunol..

[79] Hansen H.P., Trad A., Dams M., Zigrino P., Moss M., Tator M., Schon G., Grenzi P.C., Bachurski D., Aquino B. (2016). CD30 on extracellular vesicles from malignant Hodgkin cells supports damaging of CD30 ligand-expressing bystander cells with Brentuximab-Vedotin, in vitro. Oncotarget.

[80] Javidi-Sharifi N., Martinez J., English I., Joshi S.K., Scopim-Ribeiro R., Viola S.K., Edwards D.K.t., Agarwal A., Lopez C., Jorgens D. (2019). FGF2-FGFR1 signaling regulates release of Leukemia-Protective exosomes from bone marrow stromal cells. Elife.

[81] Muntion S., Ramos T.L., Diez-Campelo M., Roson B., Sanchez-Abarca L.I., Misiewicz-Krzeminska I., Preciado S., Sarasquete M.E., de Las Rivas J., Gonzalez M. (2016). Microvesicles from Mesenchymal Stromal Cells Are Involved in HPC-Microenvironment Crosstalk in Myelodysplastic Patients. PLoS ONE.

[82] Ramos T.L., Sánchez-Abarca L.I., Rosón B., Redondo A., Rodríguez C., Rodríguez H., Ortega R., Rico A., Preciado S., López Villar O. (2016). Extracellular Vesicles Play an Important Role in Intercellular Communication Between Bone Marrow Stroma and Hematopoietic Progenitor Cells in Myeloproliferative Neoplasms. Blood.

[83] Zhang B., Nguyen L.X.T., Li L., Zhao D., Kumar B., Wu H., Lin A., Pellicano F., Hopcroft L., Su Y.L. (2018). Bone marrow niche trafficking of miR-126 controls the self-renewal of leukemia stem cells in chronic myelogenous leukemia. Nat. Med..

[84] Roccaro A.M., Sacco A., Maiso P., Azab A.K., Tai Y.T., Reagan M., Azab F., Flores L.M., Campigotto F., Weller E. (2013). BM mesenchymal stromal cell-derived exosomes facilitate multiple myeloma progression. J. Clin. Investig..

[85] Dabbah M., Attar-Schneider O., Tartakover Matalon S., Shefler I., Jarchwsky Dolberg O., Lishner M., Drucker L. (2017). Microvesicles derived from normal and multiple myeloma bone marrow mesenchymal stem cells differentially modulate myeloma cells’ phenotype and translation initiation. Carcinogenesis.

[86] Deng M., Yuan H., Liu S., Hu Z., Xiao H. (2019). Exosome-transmitted LINC00461 promotes multiple myeloma cell proliferation and suppresses apoptosis by modulating microRNA/BCL-2 expression. Cytotherapy.

[87] Arendt B.K., Walters D.K., Wu X., Tschumper R.C., Jelinek D.F. (2014). Multiple myeloma dell-derived microvesicles are enriched in CD147 expression and enhance tumor cell proliferation. Oncotarget.

[88] Raimondo S., Saieva L., Corrado C., Fontana S., Flugy A., Rizzo A., De Leo G., Alessandro R. (2015). Chronic myeloid leukemia-derived exosomes promote tumor growth through an autocrine mechanism. Cell Commun. Signal.

[89] Razmkhah F., Soleimani M., Ghasemi S., Kafi-Abad S.A. (2019). MicroRNA-21 over expression in umbilical cord blood hematopoietic stem progenitor cells by leukemia microvesicles. Genet. Mol. Biol..

[90] Razmkhah F., Soleimani M., Mehrabani D., Karimi M.H., Amini Kafi-Abad S., Ramzi M., Iravani Saadi M., Kakoui J. (2017). Leukemia microvesicles affect healthy hematopoietic stem cells. Tumour Biol..

[91] Zhao C., Du F., Zhao Y., Wang S., Qi L. (2019). Acute myeloid leukemia cells secrete microRNA-4532-containing exosomes to mediate normal hematopoiesis in hematopoietic stem cells by activating the LDOC1-dependent STAT3 signaling pathway. Stem Cell Res. Ther..

[92] Abdelhamed S., Butler J.T., Doron B., Halse A., Nemecek E., Wilmarth P.A., Marks D.L., Chang B.H., Horton T., Kurre P. (2019). Extracellular vesicles impose quiescence on residual hematopoietic stem cells in the leukemic niche. EMBO Rep..

[93] Huan J., Hornick N.I., Goloviznina N.A., Kamimae-Lanning A.N., David L.L., Wilmarth P.A., Mori T., Chevillet J.R., Narla A., Roberts C.T. (2015). Coordinate regulation of residual bone marrow function by paracrine trafficking of AML exosomes. Leukemia.

[94] Hornick N.I., Doron B., Abdelhamed S., Huan J., Harrington C.A., Shen R., Cambronne X.A., Chakkaramakkil Verghese S., Kurre P. (2016). AML suppresses hematopoiesis by releasing exosomes that contain microRNAs targeting c-MYB. Sci. Signal..

[95] Cai J., Wu G., Tan X., Han Y., Chen C., Li C., Wang N., Zou X., Chen X., Zhou F. (2014). Transferred BCR/ABL DNA from K562 extracellular vesicles causes chronic myeloid leukemia in immunodeficient mice. PLoS ONE.

[96] Zhang H.M., Li Q., Zhu X., Liu W., Hu H., Liu T., Cheng F., You Y., Zhong Z., Zou P. (2016). miR-146b-5p within BCR-ABL1-Positive Microvesicles Promotes Leukemic Transformation of Hematopoietic Cells. Cancer Res..

[97] Fang Y., Garnier D., Lee T.H., D’Asti E., Montermini L., Meehan B., Rak J. (2016). PML-RARa modulates the vascular signature of extracellular vesicles released by acute promyelocytic leukemia cells. Angiogenesis.

[98] Wang B., Wang X., Hou D., Huang Q., Zhan W., Chen C., Liu J., You R., Xie J., Chen P. (2019). Exosomes derived from acute myeloid leukemia cells promote chemoresistance by enhancing glycolysis-mediated vascular remodeling. J. Cell Physiol..

[99] Taverna S., Flugy A., Saieva L., Kohn E.C., Santoro A., Meraviglia S., De Leo G., Alessandro R. (2012). Role of exosomes released by chronic myelogenous leukemia cells in angiogenesis. Int. J. Cancer.

[100] Taverna S., Amodeo V., Saieva L., Russo A., Giallombardo M., De Leo G., Alessandro R. (2014). Exosomal shuttling of miR-126 in endothelial cells modulates adhesive and migratory abilities of chronic myelogenous leukemia cells. Mol. Cancer.

[101] Umezu T., Ohyashiki K., Kuroda M., Ohyashiki J.H. (2013). Leukemia cell to endothelial cell communication via exosomal miRNAs. Oncogene.

[102] Mineo M., Garfield S.H., Taverna S., Flugy A., De Leo G., Alessandro R., Kohn E.C. (2012). Exosomes released by K562 chronic myeloid leukemia cells promote angiogenesis in a Src-dependent fashion. Angiogenesis.

[103] Ramos T.L., Sanchez-Abarca L.I., Lopez-Ruano G., Muntion S., Preciado S., Hernandez-Ruano M., Rosado B., de las Heras N., Chillon M.C., Hernandez-Hernandez A. (2015). Do endothelial cells belong to the primitive stem leukemic clone in CML? Role of extracellular vesicles. Leuk. Res..

[104] Tadokoro H., Umezu T., Ohyashiki K., Hirano T., Ohyashiki J.H. (2013). Exosomes derived from hypoxic leukemia cells enhance tube formation in endothelial cells. J. Biol. Chem..

[105] Wang J., De Veirman K., Faict S., Frassanito M.A., Ribatti D., Vacca A., Menu E. (2016). Multiple myeloma exosomes establish a favourable bone marrow microenvironment with enhanced angiogenesis and immunosuppression. J. Pathol..

[106] Hu J., Van Valckenborgh E., Menu E., De Bruyne E., Vanderkerken K. (2012). Understanding the hypoxic niche of multiple myeloma: Therapeutic implications and contributions of mouse models. Dis. Model. Mech..

[107] Umezu T., Tadokoro H., Azuma K., Yoshizawa S., Ohyashiki K., Ohyashiki J.H. (2014). Exosomal miR-135b shed from hypoxic multiple myeloma cells enhances angiogenesis by targeting factor-inhibiting HIF-1. Blood.

[108] Umezu T., Imanishi S., Azuma K., Kobayashi C., Yoshizawa S., Ohyashiki K., Ohyashiki J.H. (2017). Replenishing exosomes from older bone marrow stromal cells with miR-340 inhibits myeloma-related angiogenesis. Blood Adv..

[109] Liu Y., Zhu X.J., Zeng C., Wu P.H., Wang H.X., Chen Z.C., Li Q.B. (2014). Microvesicles secreted from human multiple myeloma cells promote angiogenesis. Acta Pharmacol. Sin..

[110] Li B., Hong J., Hong M., Wang Y., Yu T., Zang S., Wu Q. (2019). piRNA-823 delivered by multiple myeloma-derived extracellular vesicles promoted tumorigenesis through re-educating endothelial cells in the tumor environment. Oncogene.

[111] Chen Z., You L., Wang L., Huang X., Liu H., Wei J.Y., Zhu L., Qian W. (2018). Dual effect of DLBCL-derived EXOs in lymphoma to improve DC vaccine efficacy in vitro while favor tumorgenesis in vivo. J. Exp. Clin. Cancer Res..

[112] El-Saghir J., Nassar F., Tawil N., El-Sabban M. (2016). ATL-derived exosomes modulate mesenchymal stem cells: Potential role in leukemia progression. Retrovirology.

[113] Hedlund M., Nagaeva O., Kargl D., Baranov V., Mincheva-Nilsson L. (2011). Thermal- and oxidative stress causes enhanced release of NKG2D ligand-bearing immunosuppressive exosomes in leukemia/lymphoma T and B cells. PLoS ONE.

[114] Hong C.S., Muller L., Whiteside T.L., Boyiadzis M. (2014). Plasma exosomes as markers of therapeutic response in patients with acute myeloid leukemia. Front. Immunol..

[115] Jafarzadeh N., Safari Z., Pornour M., Amirizadeh N., Forouzandeh Moghadam M., Sadeghizadeh M. (2019). Alteration of cellular and immune-related properties of bone marrow mesenchymal stem cells and macrophages by K562 chronic myeloid leukemia cell derived exosomes. J. Cell Physiol..

[116] Barone M., Catani L., Ricci F., Romano M., Forte D., Auteri G., Bartoletti D., Ottaviani E., Tazzari P.L., Vianelli N. (2020). The role of circulating monocytes and JAK inhibition in the infectious-driven inflammatory response of myelofibrosis. Oncoimmunology.

[117] Haderk F., Schulz R., Iskar M., Cid L.L., Worst T., Willmund K.V., Schulz A., Warnken U., Seiler J., Benner A. (2017). Tumor-derived exosomes modulate PD-L1 expression in monocytes. Sci. Immunol..

[118] Arkhypov I., Lasser S., Petrova V., Weber R., Groth C., Utikal J., Altevogt P., Umansky V. (2020). Myeloid Cell Modulation by Tumor-Derived Extracellular Vesicles. Int. J. Mol. Sci..

[119] Bruns H., Bottcher M., Qorraj M., Fabri M., Jitschin S., Dindorf J., Busch L., Jitschin R., Mackensen A., Mougiakakos D. (2017). CLL-cell-mediated MDSC induction by exosomal miR-155 transfer is disrupted by vitamin D. Leukemia.

[120] Reiners K.S., Shatnyeva O., Vasyutina E., Bosl T., Hansen H.P., Hallek M., Herling M., von Strandmann E.P. (2017). Extracellular vesicles released from chronic lymphocytic leukemia cells exhibit a disease relevant mRNA signature and transfer mRNA to bystander cells. Haematologica.

[121] Reiners K.S., Topolar D., Henke A., Simhadri V.R., Kessler J., Sauer M., Bessler M., Hansen H.P., Tawadros S., Herling M. (2013). Soluble ligands for NK cell receptors promote evasion of chronic lymphocytic leukemia cells from NK cell anti-tumor activity. Blood.

[122] Smallwood D.T., Apollonio B., Willimott S., Lezina L., Alharthi A., Ambrose A.R., De Rossi G., Ramsay A.G., Wagner S.D. (2016). Extracellular vesicles released by CD40/IL-4-stimulated CLL cells confer altered functional properties to CD4+ T cells. Blood.

[123] Wang J., De Veirman K., De Beule N., Maes K., De Bruyne E., Van Valckenborgh E., Vanderkerken K., Menu E. (2015). The bone marrow microenvironment enhances multiple myeloma progression by exosome-mediated activation of myeloid-derived suppressor cells. Oncotarget.

[124] Borrelli C., Ricci B., Vulpis E., Fionda C., Ricciardi M.R., Petrucci M.T., Masuelli L., Peri A., Cippitelli M., Zingoni A. (2018). Drug-Induced Senescent Multiple Myeloma Cells Elicit NK Cell Proliferation by Direct or Exosome-Mediated IL15 Trans-Presentation. Cancer Immunol. Res..

[125] Vulpis E., Cecere F., Molfetta R., Soriani A., Fionda C., Peruzzi G., Caracciolo G., Palchetti S., Masuelli L., Simonelli L. (2017). Genotoxic stress modulates the release of exosomes from multiple myeloma cells capable of activating NK cell cytokine production: Role of HSP70/TLR2/NF-kB axis. Oncoimmunology.

[126] Higuchi H., Yamakawa N., Imadome K.I., Yahata T., Kotaki R., Ogata J., Kakizaki M., Fujita K., Lu J., Yokoyama K. (2018). Role of exosomes as a proinflammatory mediator in the development of EBV-associated lymphoma. Blood.

[127] Ahmed W., Philip P.S., Attoub S., Khan G. (2015). Epstein-Barr virus-infected cells release Fas ligand in exosomal fractions and induce apoptosis in recipient cells via the extrinsic pathway. J. Gen. Virol..

[128] Flanagan J., Middeldorp J., Sculley T. (2003). Localization of the Epstein-Barr virus protein LMP 1 to exosomes. J. Gen. Virol..

[129] Pegtel D.M., Cosmopoulos K., Thorley-Lawson D.A., van Eijndhoven M.A., Hopmans E.S., Lindenberg J.L., de Gruijl T.D., Wurdinger T., Middeldorp J.M. (2010). Functional delivery of viral miRNAs via exosomes. Proc. Natl. Acad. Sci. USA.

[130] Gutzeit C., Nagy N., Gentile M., Lyberg K., Gumz J., Vallhov H., Puga I., Klein E., Gabrielsson S., Cerutti A. (2014). Exosomes derived from Burkitt’s lymphoma cell lines induce proliferation, differentiation, and class-switch recombination in B cells. J. Immunol..

[131] Hansen H.P., Engels H.M., Dams M., Paes Leme A.F., Pauletti B.A., Simhadri V.L., Durkop H., Reiners K.S., Barnert S., Engert A. (2014). Protrusion-guided extracellular vesicles mediate CD30 trans-signalling in the microenvironment of Hodgkin’s lymphoma. J. Pathol..

[132] Mancek-Keber M., Lainscek D., Bencina M., Chen J.G., Romih R., Hunter Z.R., Treon S.P., Jerala R. (2018). Extracellular vesicle-mediated transfer of constitutively active MyD88(L265P) engages MyD88(wt) and activates signaling. Blood.

[133] Gheldof D., Mullier F., Bailly N., Devalet B., Dogne J.M., Chatelain B., Chatelain C. (2014). Microparticle bearing tissue factor: A link between promyelocytic cells and hypercoagulable state. Thromb Res..

[134] Gheldof D., Haguet H., Dogne J.M., Bouvy C., Graux C., George F., Sonet A., Chatelain C., Chatelain B., Mullier F. (2017). Procoagulant activity of extracellular vesicles as a potential biomarker for risk of thrombosis and DIC in patients with acute leukaemia. J. Thromb Thrombolysis.

[135] Pluchart C., Poitevin G., Colinart-Thomas M., Guimard G., Audonnet S., Terryn C., Nguyen P. (2019). Vincristine induces procoagulant activity of the human lymphoblastic leukemia cell line Jurkat through the release of extracellular vesicles. J. Thromb. Thrombolysis.

[136] Zannoni J., Mauz N., Seyve L., Meunier M., Pernet-Gallay K., Brault J., Jouzier C., Laurin D., Pezet M., Pernollet M. (2019). Tumor microenvironment and clonal monocytes from chronic myelomonocytic leukemia induce a procoagulant climate. Blood Adv..

[137] Duchemin J., Ugo V., Ianotto J.C., Lecucq L., Mercier B., Abgrall J.F. (2010). Increased circulating procoagulant activity and thrombin generation in patients with myeloproliferative neoplasms. Thromb. Res..

[138] Tan X., Shi J., Fu Y., Gao C., Yang X., Li J., Wang W., Hou J., Li H., Zhou J. (2013). Role of erythrocytes and platelets in the hypercoagulable status in polycythemia vera through phosphatidylserine exposure and microparticle generation. Thromb. Haemost..

[139] Marchetti M., Tartari C.J., Russo L., Panova-Noeva M., Leuzzi A., Rambaldi A., Finazzi G., Woodhams B., Falanga A. (2014). Phospholipid-dependent procoagulant activity is highly expressed by circulating microparticles in patients with essential thrombocythemia. Am. J. Hematol..

[140] Kissova J., Ovesna P., Bulikova A., Zavřelova J., Penka M. (2015). Increasing procoagulant activity of circulating microparticles in patients with Philadelphia-negative myeloproliferative neoplasms: A single-centre experience. Blood Coagul. Fibrinolysis.

[141] Charpentier A., Lebreton A., Rauch A., Bauters A., Trillot N., Nibourel O., Tintillier V., Wemeau M., Demory J.L., Preudhomme C. (2016). Microparticle phenotypes are associated with driver mutations and distinct thrombotic risks in essential thrombocythemia. Haematologica.

[142] Taniguchi Y., Tanaka H., Luis E.J., Sakai K., Kumode T., Sano K., Serizawa K., Rai S., Morita Y., Hanamoto H. (2017). Elevated plasma levels of procoagulant microparticles are a novel risk factor for thrombosis in patients with myeloproliferative neoplasms. Int. J. Hematol.

[143] Nielsen T., Kristensen S.R., Gregersen H., Teodorescu E.M., Christiansen G., Pedersen S. (2019). Extracellular vesicle-associated procoagulant phospholipid and tissue factor activity in multiple myeloma. PLoS ONE.

[144] Nehrbas J., Butler J.T., Chen D.W., Kurre P. (2020). Extracellular Vesicles and Chemotherapy Resistance in the AML Microenvironment. Front. Oncol..

[145] Bouvy C., Wannez A., Laloy J., Chatelain C., Dogne J.M. (2017). Transfer of multidrug resistance among acute myeloid leukemia cells via extracellular vesicles and their microRNA cargo. Leuk. Res..

[146] Peng D., Wang H., Li L., Ma X., Chen Y., Zhou H., Luo Y., Xiao Y., Liu L. (2018). miR-34c-5p promotes eradication of acute myeloid leukemia stem cells by inducing senescence through selective RAB27B targeting to inhibit exosome shedding. Leukemia.

[147] Viola S., Traer E., Huan J., Hornick N.I., Tyner J.W., Agarwal A., Loriaux M., Johnstone B., Kurre P. (2016). Alterations in acute myeloid leukaemia bone marrow stromal cell exosome content coincide with gains in tyrosine kinase inhibitor resistance. Br. J. Haematol..

[148] Yang X., Sexauer A., Levis M. (2014). Bone marrow stroma-mediated resistance to FLT3 inhibitors in FLT3-ITD AML is mediated by persistent activation of extracellular regulated kinase. Br. J. Haematol..

[149] Wang J., Li D., Zhuang Y., Fu J., Li X., Shi Q., Ju X. (2017). Exosomes derived from bone marrow stromal cells decrease the sensitivity of leukemic cells to etoposide. Oncol. Lett..

[150] Min Q.H., Wang X.Z., Zhang J., Chen Q.G., Li S.Q., Liu X.Q., Li J., Liu J., Yang W.M., Jiang Y.H. (2018). Exosomes derived from imatinib-resistant chronic myeloid leukemia cells mediate a horizontal transfer of drug-resistant trait by delivering miR-365. Exp. Cell Res..

[151] Lopes-Rodrigues V., Di Luca A., Mleczko J., Meleady P., Henry M., Pesic M., Cabrera D., van Liempd S., Lima R.T., O’Connor R. (2017). Identification of the metabolic alterations associated with the multidrug resistant phenotype in cancer and their intercellular transfer mediated by extracellular vesicles. Sci. Rep..

[152] Rajeev Krishnan S., De Rubis G., Suen H., Joshua D., Lam Kwan Y., Bebawy M. (2020). A liquid biopsy to detect multidrug resistance and disease burden in multiple myeloma. Blood Cancer J..

[153] Zhang L., Pan L., Xiang B., Zhu H., Wu Y., Chen M., Guan P., Zou X., Valencia C.A., Dong B. (2016). Potential role of exosome-associated microRNA panels and in vivo environment to predict drug resistance for patients with multiple myeloma. Oncotarget.

[154] Wang J., Hendrix A., Hernot S., Lemaire M., De Bruyne E., Van Valckenborgh E., Lahoutte T., De Wever O., Vanderkerken K., Menu E. (2014). Bone marrow stromal cell-derived exosomes as communicators in drug resistance in multiple myeloma cells. Blood.

[155] Xu H., Han H., Song S., Yi N., Qian C., Qiu Y., Zhou W., Hong Y., Zhuang W., Li Z. (2019). Exosome-Transmitted PSMA3 and PSMA3-AS1 Promote Proteasome Inhibitor Resistance in Multiple Myeloma. Clin. Cancer Res..

[156] Moloudizargari M., Abdollahi M., Asghari M.H., Zimta A.A., Neagoe I.B., Nabavi S.M. (2019). The emerging role of exosomes in multiple myeloma. Blood Rev..

[157] Feng Y., Zhong M., Zeng S., Wang L., Liu P., Xiao X., Liu Y. (2019). Exosome-derived miRNAs as predictive biomarkers for diffuse large B-cell lymphoma chemotherapy resistance. Epigenomics.

[158] Aung T., Chapuy B., Vogel D., Wenzel D., Oppermann M., Lahmann M., Weinhage T., Menck K., Hupfeld T., Koch R. (2011). Exosomal evasion of humoral immunotherapy in aggressive B-cell lymphoma modulated by ATP-binding cassette transporter A3. Proc. Natl. Acad. Sci. USA.

[159] Koch R., Aung T., Vogel D., Chapuy B., Wenzel D., Becker S., Sinzig U., Venkataramani V., von Mach T., Jacob R. (2016). Nuclear Trapping through Inhibition of Exosomal Export by Indomethacin Increases Cytostatic Efficacy of Doxorubicin and Pixantrone. Clin. Cancer Res..

[160] Tosetti F., Vene R., Camodeca C., Nuti E., Rossello A., D’Arrigo C., Galante D., Ferrari N., Poggi A., Zocchi M.R. (2018). Specific ADAM10 inhibitors localize in exosome-like vesicles released by Hodgkin lymphoma and stromal cells and prevent sheddase activity carried to bystander cells. Oncoimmunology.

[161] Liu J., Wang X. (2019). Focus on exosomes-From pathogenic mechanisms to the potential clinical application value in lymphoma. J. Cell Biochem..

[162] Bellavia D., Raimondo S., Calabrese G., Forte S., Cristaldi M., Patinella A., Memeo L., Manno M., Raccosta S., Diana P. (2017). Interleukin 3-receptor targeted exosomes inhibit in vitro and in vivo Chronic Myelogenous Leukemia cell growth. Theranostics.

