# Supplementary material for: The “Vesicular Intelligence” Strategy of Blood Cancers
Source: Genes (Basel). 2021 Mar 13;12(3):416. doi: 10.3390/genes12030416 (PMC7999060; doi:10.3390/genes12030416)
Supplement: Supplementary file 1 [file genes-12-00416-s001.pdf]

Supplementary Table S1. Summary of current studies on EVs as biomarkers

Disease	Source	Cargo	Role	Reference
AML	serum	TGF-β1, CD34, CD33, CD117	Prognostic	[21]
AML	serum	miR-150<br>miR-155<br>miR-1246	Prognostic	[23]
AML	serum	miR-125b	Prognostic	[24]
AML	serum	miR-10b	Prognostic	[25]
CLL	serum	CD19, CD37	Prognostic	[27]
CLL	plasma	S100-A9 protein	Prognostic	[29]
CLL	plasma	CD52	Prognostic	[30]
CLL	plasma	miR-150, miR-155, miR-223, miR-29<br>CD37, CD9, CD63	Diagnostic	[31]
CLL	plasma	mc-COX2	Prognostic	[33]
MF	plasma	CD61, CD62P	Prognostic	[34]
MM	serum	CD38, CD138, CD44, CD147	Prognostic	[37]
MM	serum	let-7b and miR-18a	Prognostic	[40]
nHL /HL	plasma	CD20/CD30	Diagnostic/Prognostic	[20]
Lymphoma	plasma	CD20	Prognostic	[43]
HL	plasma	miR-23p<br>miR-127-3p<br>miR-21-5p<br>miR-155-5p<br>let-7a-5p	Diagnostic	[44]
Lymphoma	plasma	BCL-6<br>c-myc	Prognostic	[45]

Supplementary Table S2. Summary of current studies on EVs: re-education of the bone marrow niche

Disease	EV origin/Source	Target	Cargo	Functional effects	Reference
AML	AML cells	MSC/stromal cells	/	Downregulating of KITL, CXCL12, IGF1; Reducing support to normal hemopoiesis	[52]
AML	AML cells	Stromal cells	miR-155 miR-375 miR-150	Reducing secretion of cytokines and growth factor; Affecting retention and differentiation of HSC in the bone marrow	[54]
AML/MDS	AML/MDS cells	MSC	miR-7977	Reducing the hemopoiesis supportive capacity	[55]
CLL	CLL cells	MSC	miR-202-3p	Promoting migration, survival and proliferation	[32]
CLL	Plasma	Stromal cells	/	Production of VEGF, promoting survival of B cells	[26]
CLL	CLL cells	Stromal cells	miR-146a miR-451	Favoring transition toward cancer-associated fibroblasts	[58]
CML	CML cells	Stromal cells	/	IL-8 production, promoting CML cell function	[59,60]
CML	CML cells	MSC	/	Increasing the expression of TGF-β1 with the promotion of CML cell proliferation	[61]
CML	CML cells	MSC	miR-711	Weakening the adhesive ability	[62]
CML	CML cells	MSC	miR-320	Inhibiting osteogenesis	[63]
MM	MM cells	MSC	/	Suppressing osteoblastic differentiation ability	[68]
MM	MM cells	MSC	lncRNA RUNX2-AS1	Repressing osteogenesis	[71]
MM	Stromal cells	MM cells	miR-10a	Inhibition of cell proliferation	[72]
MM	MM cells	MSC	miR-146a	Inducing the secretion of cytokines and promoting MM cell viability and migration	[73]
HL	HL cells	Fibroblasts	/	Inducing inflammatory phenotype and supporting tumor growth	[76]

Supplementary Table S3. Summary of current studies on EVs: signals from the microenvironment

Disease	EV origin	Target	Cargo	Ffunctional effects	Reference
AML	Stromal cells	AML cells	FGF-2	Protecting from TKIs	[78]
MPN	MSC	MPN CD34+ cells	miR-155	Promoting clonogenic ability	[80]
CML	Endothelial cells	CML leukemic stem cells	miR-126	Supporting quiescence and leukemia growth	[81]
CLL	MSC	CLL B cells	/	Promoting viability and chemoresistance	[57]
MM	MSC	MM cells	LINC00461	Promoting proliferation	[84]
MM	MSC	MM cells	miR-15a	Promoting MM cell growth	[82]

Supplementary Table S4. Summary of current studies on EVs: normal hemopoiesis restrain/transformation

Disease	EV origin/source	Target	Cargo	Functional effect	Reference
AML	AML cells	CD34+ cells	miR-4532	Repressing normal hemopoietic function	[89]
AML	AML cells/plasma	Long-Term hemopoietic stem cells	miR-1246	Inducing quiescence and eliciting DNA damage	[90]
AML	AML cells/plasma	Hemopoietic stem cells	miR-150, miR-155	Suppressing hemopoietic function by inhibiting c-MYB	[92]
CML	CML cells	/	BCR-ABL	Promoting in vivo development of CML	[93]
CML	CML cells	Mononuclear cells	miR-146b-5p	Promoting leukemic transformation	[94]

Supplementary Table S5. Summary of current studies on EVs: angiogenesis promotion

Disease	EV origin	Target	Cargo	Functional effects	Reference
AML	Acute Promyelocytic Leukemia cells	Endothelial cells	Tissue factor, VEGF, IL-8	Endothelial stimulating activity	[95]
AML	AML cells	Endothelial cells	VEGF, VEGFR	Promoting vascular remodeling	[96]
CML	CML cells	Endothelial cells	miR-126<br>miR-92a	Increasing survival, motility and vascular tube formation	[97,98,99]
MM	Hypoxic MM cells	Endothelial cells	miR-135b	Promoting angiogenesis	[105]
MM	MM cells	Endothelial cells	Piwi-interacting RNA-823	Promoting proliferation, tube formation and migration	[108]
Lymphoma	Lymphoma cells	Endothelial cells<br>Stromal cells	Angiogenic proteins including VEGF	Stimulating angiogenesis	[109]
Lymphoma	T-cell leukemia/lymphoma	MSC	miR-21, miR-155, VEGF	Increasing proliferation and angiogenic markers expression	[110]

Supplementary Table S6. Summary of current studies on EVs: immune evasion

Disease	EV origin/source	Target	Cargo	Functional effects	Reference
AML	Serum	Normal NK cells	/	Decreasing toxicity and NKG2D expression	[21]
MF	Monocytes	/	Inflammatory cytokines	Promoting Ruxolitinb-driven inflammatory signaling	[116]
CML	CML cells	MSC	/	Modulating the inflammatory molecules (TNF-alpha and NO) and the redox potential	[115]
CLL	Monocytes	/	miR-155	Promoting differentiation into MDSC	[119]
CLL	CLL cells	CD4+ T cells	miR-363	Promoting migration, immunological signaling, interactions with tumor cells	[122]
CLL	CLL cells	Monocytes/ fibroblasts	mRNA	Spreading tumor signaling (splicing factors, the TCL1A oncogene, and tyrosine kinases) within the microenvironment	[120]
MM	Stromal cells	MDSCs	/	Promoting MDSC survival and suppressive activity on T cells	[123]
Lymphoma	Lymphoma cells	NK cells	NKG2D ligand MICA/B and ULBP1/2	Inhibiting NK cell cytotoxicity	[113]
Lymphoma	EBV-associated lymphoma cells	Monocytes/ macrophages	EBV-derived non-coding RNAs such as BART miR	Inducing an immune regulatory phenotype	[126]
Lymphoma	HL cells	Immune cells	CD30 receptor	Educating distant immune cells stimulating their IL-8 release	[131]
Lymphoma	B cells	Mast cells and macrophages	MYD88	Activating pro-inflammatory signaling	[132]

Supplementary Table S7. Summary of current studies on EVs: hypercoagulability

Disease	EV origin/source	Target	Cargo	Functional effects	Reference
AML	AML cells	/	TF and PS	Promoting procoagulant activity	[133]
CMML	CMML monocytes	Normal MSC	TF	Promoting procoagulant activity	[136]
MPN	plasma	/	TF	Biomarker of thrombotic events	[142]

Supplementary Table S8. Summary of current studies on EVs: drug resistance

Disease	EV origin/source	Target	Cargo	Functional effects	Reference
AML	Chemoresistant AML cells	Chemosensitive AML cells	miR-19b, miR-20a	Inducing expression chemoresistance biomarkers	[145]
AML	Stromal cells	AML cells	miR-155, miR-375 TGF-β1	Inducing chemoresistance	[147]
AML	Leukemic stem cells	/	miR-34c-5p	Favoring chemoresistance and senescence driven-eradication of leukemic stem cells	[146]
CML	Imatinib-resistant CML cells	Imatinib sensitive CML cells	miR-365	Conferring drug resistance	[150]
CML	Chemoresistant CML cells	Chemosensitive CML cells	P-gp	Inducing metabolic switch towards multidrug resistance phenotype	[151]
MM	MM cells/plasma	/	miR-16, miR-15a, miR-20a, miR-17	Biomarkers of resistance to Bortezomib	[153]
MM	Stromal cells	MM cells	/	Inducing drug resistance to Bortezomib	[154]
MM	MSC	MM cells	PSMA3 and PSMA3-AS1	Transmitting proteasome inhibitor resistance	[155]
Lymphoma	DLBCL serum	/	miR-99a, miR-125b	Correlating to chemoresistance	[157]
Lymphoma	B- cells	/	CD20	Capturing Rituximab and constraining therapeutic effectiveness	[158]
Lymphoma	Lymphoma cells	/	ADAM10	Interfering with immunotherapy	[160]
